# Mapping the citation network on vitamin D research in Australia: a data-driven approach

**DOI:** 10.3389/fmed.2023.1298190

**Published:** 2023-11-28

**Authors:** Belinda Neo, Xiaochen Qu, Eleanor Dunlop, Carrington Shepherd, Erin I. Walsh, Nicolas Cherbuin, Lucinda J. Black

**Affiliations:** ^1^Curtin Medical School, Curtin University, Bentley, WA, Australia; ^2^Curtin School of Population Health, Curtin University, Bentley, WA, Australia; ^3^Institute for Physical Activity and Nutrition (IPAN), School of Exercise and Nutrition Sciences, Deakin University, Burwood, VIC, Australia; ^4^Telethon Kids Institute, The University of Western Australia, Nedlands, WA, Australia; ^5^Ngangk Yira Research Institute for Change, Murdoch University, Murdoch, WA, Australia; ^6^Population Health Exchange, National Centre for Epidemiology and Population Health, Australian National University, Canberra, ACT, Australia; ^7^Department of Health Economics Wellbeing and Society, National Centre for Epidemiology and Population Health, Australian National University, Canberra, ACT, Australia

**Keywords:** citation network analysis, data-driven approach, literature map, vitamin D, Australia

## Abstract

Vitamin D research can vary geographically, as vitamin D status is influenced by latitude, season, dietary intake, body mass index, ethnicity, and public health initiatives. Over the last two decades, research on vitamin D has increased in Australia, where the potential for sun exposure (a major source of vitamin D) is high. We aimed to identify key topics and gaps in vitamin D research in Australia using a data-driven approach. A literature search limited to Australian studies was conducted in the Web of Science Core Collection database. Citation network analysis was conducted to identify clusters and sub-clusters, depicted using word clouds. Topic analysis of each cluster and sub-cluster was conducted to identify topics and sub-topics, respectively. From 934 publications (over the period 1984–2022), nine topics and 60 sub-topics were identified. The nine topics were: vitamin D in vulnerable populations and its impact on child development; impact of sun exposure and ultraviolet-B radiation on various health conditions; vitamin D and falls and fractures in older adults; vitamin D and its association with health outcomes; vitamin D from sun exposure; testing of vitamin D status in Australia; vitamin D, calcium, and musculoskeletal health; vitamin D status and knee osteoarthritis; and vitamin D status and exercise performance in athletes. There were limited publications on vitamin D in Aboriginal and Torres Strait Islander peoples and dietary vitamin D. We have provided an overview of vitamin D research in Australia. The research trends and knowledge gaps identified can guide future research to better inform public health initiatives in Australia.

## 1 Introduction

Vitamin D is a fat-soluble vitamin that is essential for bone health. Vitamin D can be obtained from various sources such as ultraviolet-B (UVB) radiation from sun exposure, dietary intake of foods that contain vitamin D (e.g., fish, eggs, and meat), and supplements (1, 2). There are two major forms of vitamin D, vitamin D_3_ (cholecalciferol), which can be obtained via sun exposure and dietary sources, and vitamin D_2_ (ergocalciferol), obtained from dietary sources. These two forms of vitamin D are converted in the body to 25-hydroxyvitamin D (25(OH)D), the main circulating form of vitamin D in the body and the form used as a marker of vitamin D status (1).

Vitamin D status can be influenced by latitude, season (e.g., summer versus winter), food and supplement intake habits, body mass index, ethnicity, and public health initiatives (e.g., SunSmart®, a long-running skin cancer prevention program by Cancer Council Australia) (3–5). Due to the specificity of vitamin D sources, the research conducted in each country is unique to its population. In Australia, knowledge of vitamin D has progressed considerably over the last decade. Recent research has provided new insights into the vitamin D composition of foods (2, 6) and usual vitamin D intakes in Australia (7). Vitamin D intake was found to be low (<3.5 μg/day) in the general Australian population (7). Despite being a country with relatively high UVB radiation, vitamin D deficiency (defined as serum 25(OH)D concentration < 50 nmol/L) (4) is still prevalent in the general Australian population (17% of adolescents aged 12–17 years, 32% of young adults aged 18–24 years, and 20% of adults aged ≥25 years) (8, 9) and Aboriginal and Torres Strait Islander peoples (27% of adults aged ≥18 years) (10).

The role of vitamin D in human health is multifaceted, which is reflected in the wide range of research conducted to understand the metabolism, function, and requirements of vitamin D. Internationally, systematic reviews and meta-analyses on vitamin D have covered a broad range of topics on vitamin D deficiency and vitamin D health-related conditions such as rickets, osteoporosis, cancer, metabolic conditions (e.g., diabetes), mental health, and all-cause mortality (11–15). These systematic reviews and meta-analyses are highly specialised, addressing a specific area of research, but the screening of publications and synthesis of information requires a manual process that can be time-consuming to conduct (16). Furthermore, none have provided a comprehensive overview of vitamin D research specific to Australia.

Comparatively, literature mapping uses a data-driven approach to efficiently synthesise a large body of literature, removing the need for manual selection of publications (16). The data gathered through the literature search is used to drive the analysis, and publications with direct citation links (e.g., an article cited by another article) are clustered together based on their research areas (17). The final literature map provides a holistic overview of the research topics and identifies trends and potential knowledge gaps in research over time.

Vitamin D research is unique in each country due to the environment and lifestyle habits of the population. By mapping the research in Australia, we can identify population-specific evidence and areas that can be filled by future research to support tailored policies and practices. To our knowledge, the literature on vitamin D research in Australia has not been mapped using a data-driven approach. This study aims to identify key topics and knowledge gaps in vitamin D research conducted in Australia through a data-driven approach.

## 2 Methods

**Figure 1:**
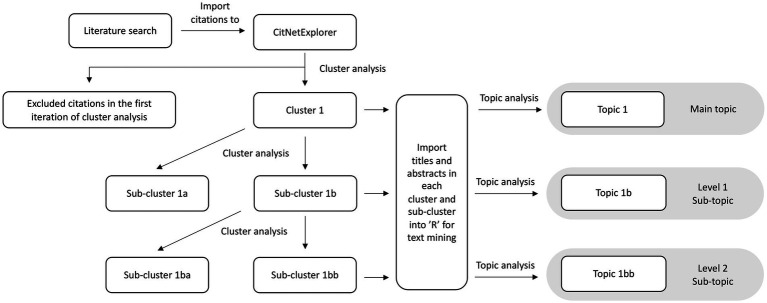
A step-by-step overview of the data-driven approach: from literature search to topics and sub-topics. Clusters consist of publications grouped through direct citation links. After the topic analysis, clusters form topics and sub-clusters form sub-topics.

The literature was mapped using the data-driven approach developed by Walsh and Cherbuin (16) to identify citation networks, a network of citations linked to other citations by direct citation link, in vitamin D research in Australia. There were three main steps for this citation network analysis, (i) systematic literature search and cluster analysis, (ii) pre-processing of text for text mining, and (iii) text mining for topics. An overview of the data-driven approach is shown in Figure 1.

### 2.1 Systematic literature search and cluster analysis

The Web of Science Core Collection database was used for the literature search conducted on the 25th August 2022. The search strategy included the following key terms in a topic search (“Vitamin D” OR “25(OH)D” OR “25-hydroxyvitamin D” OR “serum 25-hydroxyvitamin D” OR ergocalciferol OR cholecalciferol OR calcitriol OR calcifediol OR “1,25-dihydroxycholecalciferol” OR “24,25-dihydroxycholecalciferol”) AND (Australia* OR “Western Australia” OR “Northern Territory” OR Tasmania OR “New South Wales” OR Victoria OR Queensland OR “Australian Capital Territory” OR Melbourne OR Sydney OR Adelaide OR “South Australia” OR Canberra OR Brisbane OR Perth OR Hobart OR Darwin). Topic search function was used to search the key terms in the title, abstract, author keywords and keywords plus, a function in the Web of Science Core Collection database that creates index terms from common words or phrases in the reference list of a publication (18). The search was limited to articles and reviews published in the English Language. The full citation data of each publication, which includes authors, title, abstract, source, digital object identifier, and year of publication, were extracted from the database and imported into CitNetExplorer. The CitNetExplorer software helps to visualise and analyse citation networks by identifying direct citation links (19).

The imported publications underwent a cluster analysis, where the publications were clustered into groups based on direct citation links: a publication cited by another publication was clustered together by the CitNetExplorer software based on their research areas (17). There was no overlap of publications in one cluster. Each cluster repeated this step until it could not be further divided into smaller clusters. The minimum cluster size for each cluster was limited to seven publications. Publications in clusters and sub-clusters were then extracted for pre-processing.

### 2.2 Pre-processing of text for text mining

The title and abstract of publications in each cluster and sub-clusters were imported into the R statistical software (version 2022.12.0 + 353) to undergo pre-processing for text mining (20). They were converted into a text corpus (collection of a large unstructured text document) using the “tm” package (version 0.7–8) (21, 22). The text corpus was processed using lowercase conversion, spelling correction (removal of contractions and abbreviations), nonalphabetical character removal (numbers, punctuations, and special characters), stop words removal (common words that provide little meaning, e.g., “a,” “the,” and “an”), and stemming (removing word suffixes) to increase the statistical power of the results (21). High-frequency words in the corpus, which include abbreviations of vitamin D and its vitamers [e.g., “vitamin,” “25(OH)d,” “25hydroxyvitamind,” and “125dihydroxyvitamind”] were also removed from the text to elucidate topic words during text mining. The text copra was converted into a term-document matrix, where the frequency of terms was shown across the clusters and exported into spreadsheets. The frequency of terms was depicted using word clouds generated using the “wordcloud” package (version 2.6) (23), to provide a visual overview of the data from the clusters and sub-clusters. A comparison cloud was used to compare the terms in main clusters and the included and excluded publications.

### 2.3 Text mining for topics

Text mining was conducted using an open vocabulary mining technique to extract topics from the clusters and sub-clusters (21). The text mining analysis applied Latent Dirichlet Allocation (LDA) to generate topic models using the “topicmodels” package (version 0.2–12) (21, 24, 25). LDA is a statistical model in natural language parsing which extracts topic words that best describes the documents (24). Topic words are words that have the highest association with a particular topic. Documents are assigned a topic or topics based on the frequency and distribution of words that best describe the documents. The top 10 topic words of each topic or topics assigned to each cluster and sub-cluster were used in the topic analysis to create the title for each topic and sub-topic, respectively.

### 2.4 Identifying knowledge gaps

The knowledge gaps were identified through two methods: (i) sub-topics that had a low number of publications (*n* = 9); and (ii) analysing publications that were excluded in the first iteration of the clustering process. We used the 25th percentile of the total number of publications in each sub-topic to identify sub-topics that had low number of publications. The 25th percentile (*n* = 9) was used instead of a lower percentile, such as the 10th percentile (*n* = 7), to include sub-topics above the minimum cluster size (*n* = 7). The publications were excluded as they had not cited enough publications or were not cited enough to meet the minimum cluster size (*n* = 7) to be included. These publications might be relevant to vitamin D research in Australia but might be from different disciplines or obscure journals. To identify knowledge gaps, the excluded publications were analysed by creating word clouds to depict the frequency of terms, and a comparison cloud was used to depict the terms that were more prominent in the excluded publications.

## 3 Results

**Figure 2:**
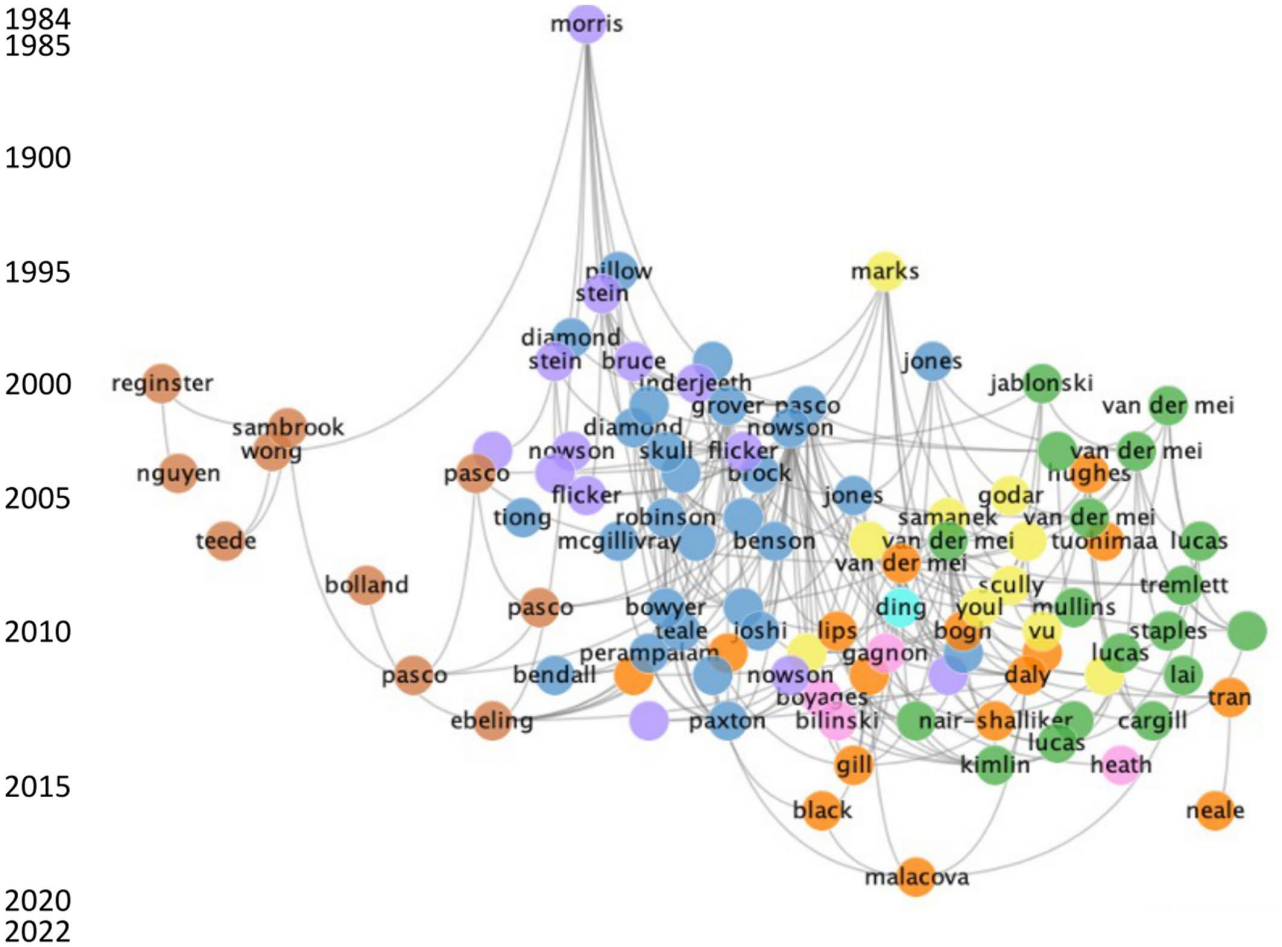
Overview of the top 100 direct citation links for vitamin D research in Australia. Each cluster is represented by a specific colour: blue, cluster 1; green, cluster 2; purple, cluster 3; light orange, cluster 4; yellow, cluster 5; dark orange, cluster 6; pink, cluster 7; light blue, cluster 8; light green, cluster 9. The gray line represents a citation link. The left side of the graph provides a label for every 5 years and the earliest and latest year of publication.

The initial literature search identified 934 publications; 675 publications were retained after the first iteration of cluster analysis (Supplementary File S1). An overview of the citation network with the top 100 citation links are shown in Figure 2. The publications included in the literature map were published from 1984 to 2022, with visibly more articles published after 2000. The earliest publication investigated the association between vitamin D status and femoral neck fractures in South Australian women (26). The most cited publication was a position paper on vitamin D and health in Australia and New Zealand, with 80 citation links (4).

### 3.1 Frequency of words within clusters

**Figure 3:**
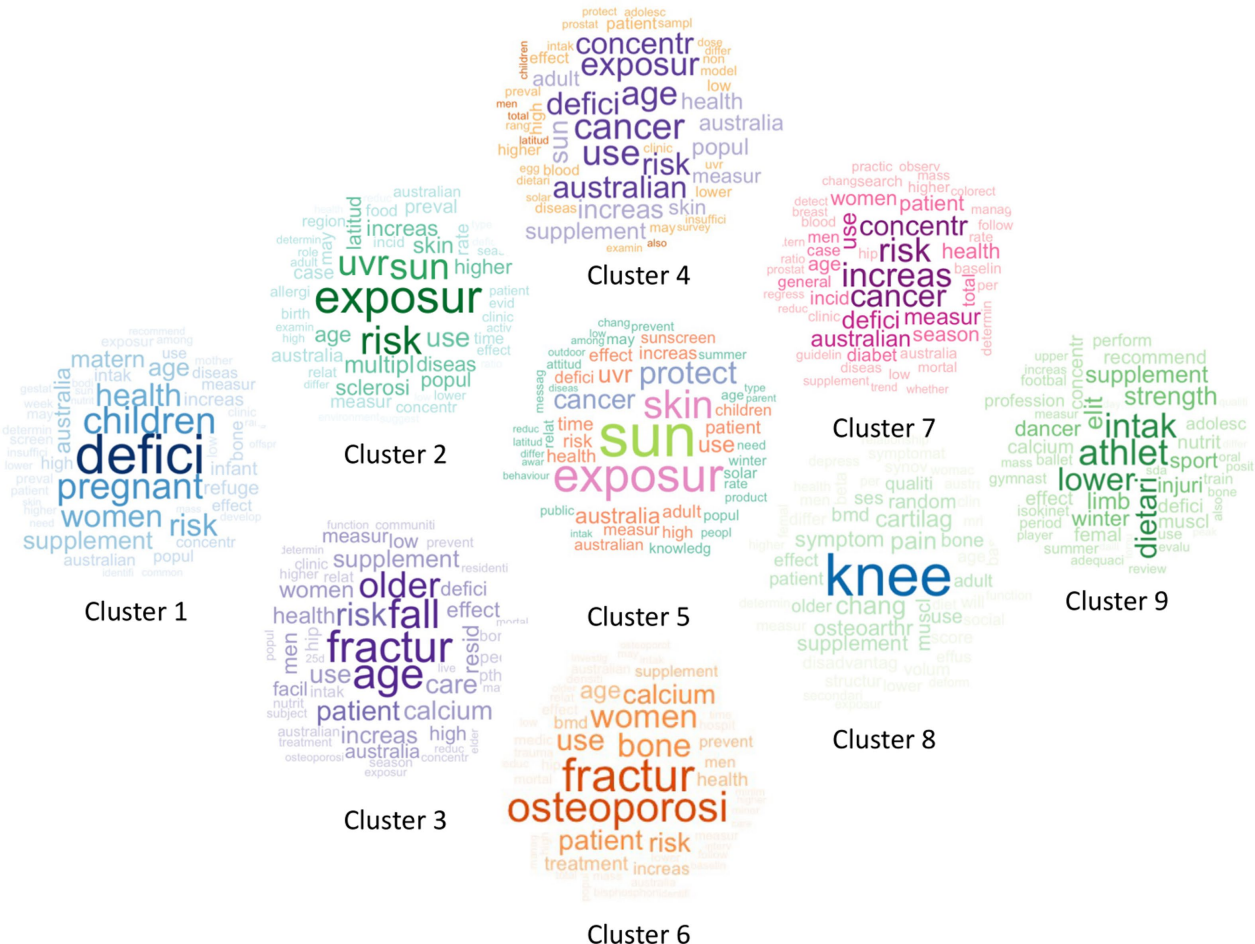
Overview of nine main clusters of vitamin D research in Australia depicted using word clouds. The word size and shade of the color represent word frequency within each cluster. More frequent words are larger and darker, while less frequent words are smaller and lighter.

A word cloud overview of the nine clusters is shown in Figure 3. The word clouds provide a brief insight into the topic of each cluster based on the frequency of words. Frequently mentioned words have a bigger font size compared to words with lower frequency. Words that were frequently mentioned in the main clusters included “deficiency,” “fall,” “fracture,” “sun,” “exposure,” “osteoporosis,” “cancer,” “knee,” and “dietary intake.” There was an overlap of words between some clusters, which included “fracture,” “fall,” “cancer,” “sun,” and “exposure”. A comparative word cloud of the main clusters can be found in Supplementary Figure S1.

### 3.2 Topic analysis

**Figure 4:**
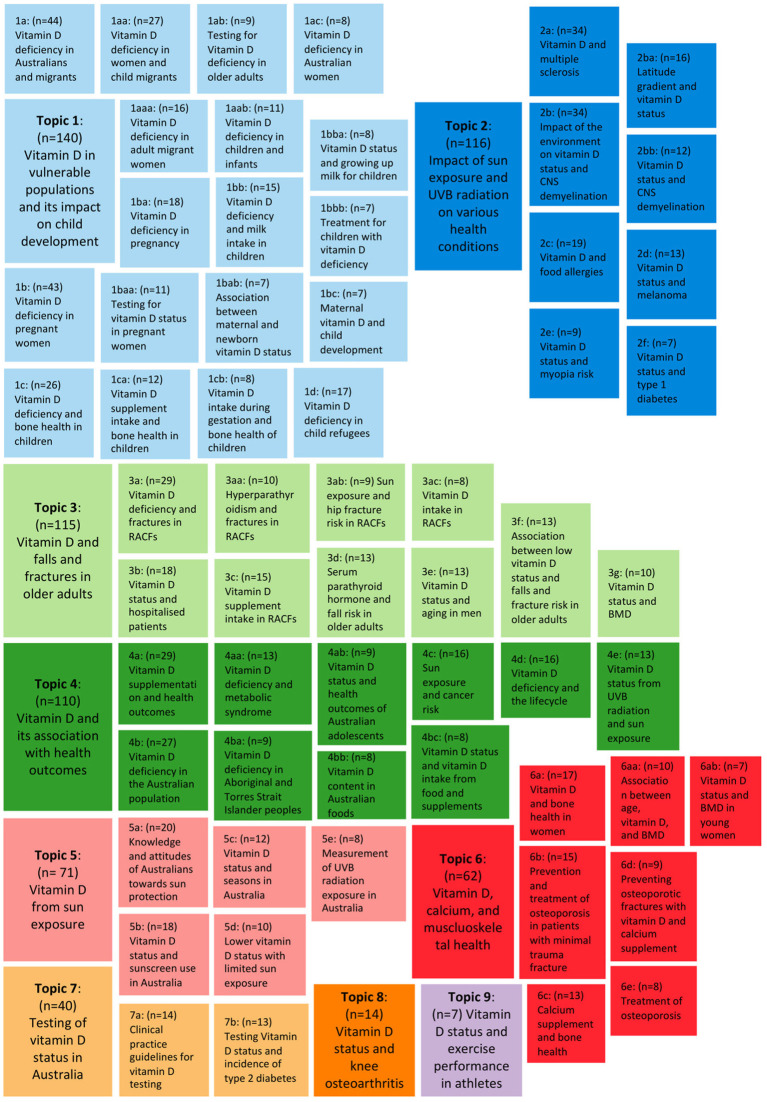
Literature map of topics and sub-topics of vitamin D research in Australia. Each topic is represented by a specific colour: light blue, cluster 1; dark blue, cluster 2; light green, cluster 3; dark green, cluster 4; pink, cluster 5; red, cluster 6; light orange, cluster 7; orange, cluster 8; purple, cluster 9. Alphabetical designations (e.g., 1a, 1aa, etc.) show the relationship between topics and sub-topics. Sub-topics are nested beside the main topic in a smaller box of the same colour. Publications excluded at each iteration are not included in this literature map, resulting in a difference between the number of publications in the main topic and the sum of their respective sub-topics. BMD, bone mineral density; CNS, central nervous system; RACFs, residential aged care facilities.

The overview of vitamin D research in Australia is shown in Figure 4, which consists of nine main topics and 60 sub-topics. The details of the topic analysis for each topic and sub-topic can be found in Supplementary Tables S1–S9.

Topic 1 had the highest number of publications (*n* = 140) and focused mainly on vitamin D in vulnerable populations and its impact on child development in Australia. There were four level one sub-topics that were categorised based on population groups, 1a: Australians and migrants; 1b: pregnant women; 1c: children; and 1d: child refugees.

Topic 2 (*n* = 116) focused on the impact of sun exposure and UVB radiation on various health conditions in Australia, which included 2a: multiple sclerosis (MS); 2b: central nervous system (CNS) demyelination; 2c: food allergies; 2d: melanoma; 2e: myopia; and 2f: type 1 diabetes. The level two sub-topics of sub-topic 2b included 2ba: latitude gradient and vitamin D status and 2bb: vitamin D status and CNS demyelination.

Topic 3 (*n* = 115) focused on vitamin D status and falls and fractures in older adults that were residents of aged care facilities (RACFs) or hospitalised patients. The first and second levels of sub-topics included predictors of falls and, or fractures in older adults, 3a: vitamin D deficiency; 3aa: hyperparathyroidism; 3ab: sun exposure in RACFs; 3 ac: vitamin D intake in RACFs; 3b: hospitalisation; 3c: vitamin D supplement intake; 3d: serum parathyroid hormone; 3e: aging in men; 3f: low vitamin D status; and 3 g: bone mineral density (BMD).

Topic 4 (*n* = 110) focused on vitamin D and its association with health outcomes, which included 4a: vitamin D supplementation and health outcomes; 4b: vitamin D deficiency in the Australian population; 4c: sun exposure and cancer risk; 4d: vitamin D deficiency in the lifecycle; 4e: vitamin D status from UVB radiation and sun exposure. There were five level two sub-topics which included 4aa: vitamin D deficiency and metabolic syndrome; 4ab: vitamin D status and health outcomes of Australian adolescents; 4ba: vitamin D deficiency in Aboriginal and Torres Strait Islander peoples; 4bb: vitamin D content in Australian foods; and 4bc: vitamin D status and vitamin D intake from food and supplements.

Topic 5 (*n* = 71) focused on vitamin D from sun exposure with only one level of sub-topics. The sub-topics expanded on various factors influencing sun exposure which included 5a: knowledge and attitudes of Australians towards sun protection; 5b: sunscreen use in Australia; 5c: seasons in Australia; 5d: lower vitamin D status with limited sun exposure; and 5e: measurement of UVB radiation exposure in Australia.

Topic 6 (*n* = 62) focused on vitamin D and calcium in musculoskeletal health in population groups (6a: women; 6b: patients with minimal trauma fracture), supplement intake (6c: calcium supplement; 6d: vitamin D and calcium supplement), and treatment (6e: treatment of osteoporosis). Sub-topic 6a is further split into two sub-topics 6aa: association between age, vitamin D, and BMD, and 6ab: vitamin D status and BMD in young women.

Topic 7 (*n* = 40) focused on the testing of vitamin D status in Australia. The two sub-topics in this topic were 7a: clinical practice guidelines for vitamin D testing and 7b: testing vitamin D status and incidence of type 2 diabetes.

Topics 8 (*n* = 14) and 9 (*n* = 7) were the only main topics without sub-topics. Topic 8 included publications on vitamin D status and knee osteoarthritis. Topic 9 had a low number of publications on vitamin D status and exercise performance in athletes, highlighting a potential knowledge gap.

The knowledge gaps were identified through sub-topics that had a low number of publications (*n* = 9). There were a low number of publications on vitamin D status in some population groups, which include women (1 ac, *n* = 8; 6ab, *n* = 7), pregnant women (1bab, *n* = 7; 1bc, *n* = 7), children (1bba, *n* = 8; 1bbb, *n* = 7), adolescents (4ab, *n* = 9), older adults (1ab, *n* = 9) and, Aboriginal and Torres Strait Islander peoples (4ba, *n* = 9). There were also a low number of publications on vitamin D and some health outcomes, which include myopia risk (2e, *n* = 9), type 1 diabetes (2f, *n* = 7), sun exposure and hip fracture risk in RACF (3ab, *n* = 9), preventing osteoporotic fractures with vitamin D and calcium supplement (6d, *n* = 9), and treatment of osteoporosis (6e, *n* = 8). Lastly, there were limited publications in dietary vitamin D (1cb, *n* = 8; 3 ac, *n* = 8; 4bb, *n* = 8; 4bc, *n* = 8), and measurement of UVB radiation exposure in Australia (5e, *n* = 8).

### 3.3 Year of publications

**Figure 5:**
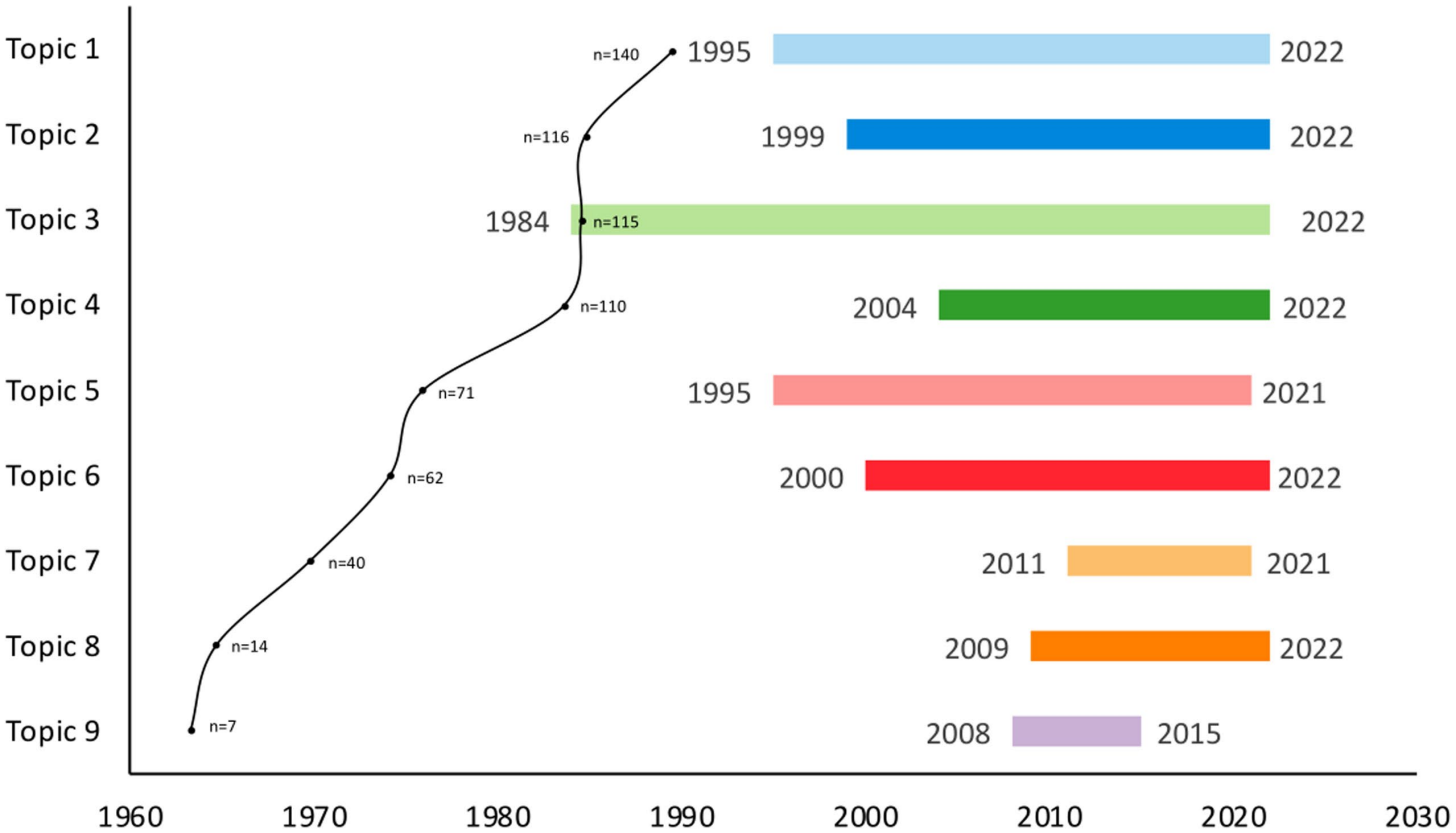
Range of publication year and number of publications for each topic. The range of publication years of each topic is represented by a specific colour: light blue, topic 1; dark blue, topic 2; light green, topic 3; dark green, topic 4; pink, topic 5; red, topic 6; light orange, topic 7; dark orange, topic 8; purple, topic 9. A dot on the graph represents the number of publications in each topic, and the number of publications is provided next to the dot.

The range of publications years and number of publications for each topic is shown in Figure 5. The topic with the largest range was topic 3, which included publications from 1984 to 2022. Most of the topics are current as their most recent year of publication are within the last 2 years (2021–2022), except for topic 9, with the most recent year of publication in 2015. Topic 9 also had the lowest range of publication years (7 years) with the lowest number of publications (*n* = 7).

### 3.4 Excluded publications

**Figure 6:**
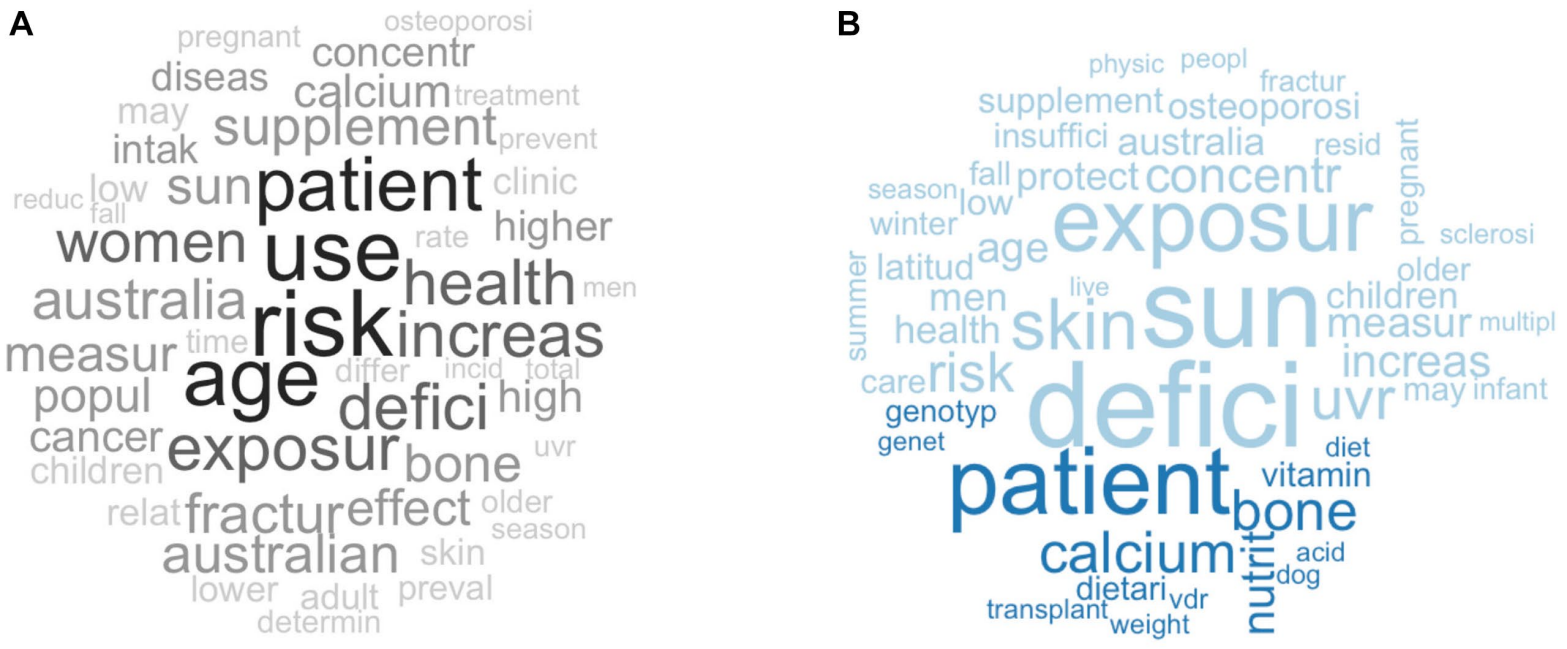
**(A)** A word cloud of the excluded publications from the first iteration of the clustering process; **(B)** A comparison word cloud of included (light blue) and excluded (dark blue) publications. The word size and shade of the colour represent word frequency within each cluster. More frequent words are larger and darker, while less frequent words are smaller and lighter.

The publications excluded in the first iteration of clustering were analysed for frequency of words (Figure 6A). The most frequent words were “patient,” “use,” “risk,” and “age” which were also shown in the main cluster word clouds. A comparison cloud was used to elicit unique words from the included and excluded publications (Figure 6B). The comparison cloud showed that more unique words were covered in the included publications. There were also overlapping words such as “patient,” “bone,” and “calcium” that were also identified in the included publications (Figure 3), but a higher frequency of those words was identified in the excluded publications (Figure 6B). This suggests that the research surrounding those areas might have been more prominent in the excluded publications than in the included publications. New unique words that were not depicted in the initial word cloud of the excluded publications were also revealed, which include, “VDR” (vitamin D receptor), “genetics,” and “genotype.”

## 4 Discussion

Using a data-driven approach, we efficiently synthesised a large amount of literature and categorised them into topics and sub-topics to elucidate research trends and knowledge gaps. The amount of research on vitamin D in Australia has increased considerably since the 2000s, with the earliest publication in 1984. The main topics were clustered based on direct citation links which resulted in overlapping content between some topic areas. Our analysis revealed common topics which included vitamin D deficiency, vitamin D status and health outcomes, falls and fractures in older adults, sun exposure, and testing of vitamin D status in Australia.

Vitamin D deficiency was a common topic identified across two main topics, vitamin D in vulnerable populations and its impact on child development (Topic 1, *n* = 140) and vitamin D and its association with health outcomes (Topic 4, *n* = 110). This is not surprising as vitamin D deficiency is prevalent in Australia (8, 9, 27). Both topics focused on vitamin D deficiency in specific population groups, which include pregnant women, children, older adults, migrants, and Aboriginal and Torres Straits Islander peoples. Research on vitamin D deficiency by age group is of interest due to its impact on growth, development, and musculoskeletal function. Maternal vitamin D status is crucial for foetal development and is linked to the birth weight and bone mass of neonates (28). In children, vitamin D deficiency may affect musculoskeletal development resulting in rickets (29). Vitamin D deficiency in older adults, especially postmenopausal women, may increase the risk of falls, fractures, and osteoporosis (30, 31). In postmenopausal women, vitamin D deficiency may increase the secretion of parathyroid hormone, which increases cortical bone porosity and fracture risk (31). Vitamin D deficiency is also prevalent in Aboriginal and Torres Strait Islander peoples, 27% were found to be deficient, and the percentage increases to 39% for those living in remote areas (10). Hence, strategies to mitigate vitamin D deficiency, particularly in these population groups, are crucial to prevent deficiency-related health outcomes.

Another common topic identified in the literature map is vitamin D and various health outcomes in topics 2 (*n* = 116) and 4 (*n* = 110). The health outcome that had the highest number of publications within these two topics is found in topic 2, on vitamin D in MS (sub-topic 2a, *n* = 34) and CNS demyelination (sub-topic 2b, *n* = 34). MS is an immune-mediated neurological condition characterised by CNS demyelination. The prevalence of MS in 2021 was about 33,000 people, an increase of about 7,700 people since 2017 (32). Vitamin D deficiency is associated with the risk of developing MS (33, 34). The high amount of research collated in these two groups may be attributed in part to the Australian Multi-centre Study of Environment and Immune Function (the Ausimmune Study) (35) and the Southern Tasmanian Multiple Sclerosis Longitudinal Study (36). These population studies investigate associations between environmental factors, including some vitamin D-related factors (e.g., UVB radiation, changes in season), and MS onset or MS progression. The broad latitudinal gradient of Australia allows MS researchers to investigate the impact of these environmental changes within a single population. For example, Hobart, Tasmania, has a higher latitude (42.5°S) and a higher prevalence of MS compared to Newcastle, New South Wales, with a lower latitude (32.9°S) (37). The unique environment of Australia and the links between vitamin D and MS has increased the amount of vitamin D and MS research conducted in Australia.

Falls and fractures related to vitamin D status in older adults have also been widely researched in Australia (topic 3, *n* = 115). Among older adults in Australia, falls are the primary reason for hospitalisation due to injuries and death due to injuries, and 50% of hospitalised fall injuries were fractures (38). Older adults residing in RACFs (sub-topic 3a, *n* = 29; 3aa, *n* = 10; 3ab, *n* = 9; 3 ac, *n* = 8; 3c, *n* = 15) or in hospitals (sub-topic 3b, *n* = 18) were of interest due to the limited sunlight exposure in these facilities which decreases the opportunity for cutaneous vitamin D synthesis (39). Vitamin D supplementation (≥800 IU/day) has been recommended to mitigate vitamin D deficiency in RACFs in Australia, but integration to practice has been slow (40, 41). Hence, ViDAus, an intervention study, was conducted in New South Wales and South Australia to assess the feasibility of stepwise education and monitoring of vitamin D supplementation in RACFs (41, 42). The results of the intervention showed a small significant improvement in vitamin D supplementation at 12 months (3.9%) and 18 months (4.6%), which might be due to individuals already consuming a vitamin D supplement at baseline (56.2%). However, as ViDAus recruited participants through convenience sampling in two Australian states, it was not representative of the Australian population. Future nationwide interventions are required to provide feasible guidelines for implementing vitamin D supplementation in RACFs to prevent falls and fractures.

There was a particular focus on sun exposure (topic 5, *n* = 71; sub-topic 4c, *n* = 16; sub-topic 4e, *n* = 13) as much of Australia is geographically situated at a relatively low latitude which predisposes many regions to high levels of solar UVB radiation (43). Due to the high risk of skin cancer from sun exposure, sun safety has been widely advocated as a public health initiative through the SunSmart® campaign (3). Australians are encouraged to wear protective clothing and sunscreen to protect against UV radiation, potentially limiting cutaneous vitamin D production. There are currently no specific guidelines on safe sun exposure for optimal vitamin D status in Australia. However, an ongoing multi-centre randomised controlled trial, the Sun Exposure and Vitamin D Supplementation (SEDS) Study, is testing scenarios of safe sun exposure with and without vitamin D supplementation on vitamin D status (44). The results of the study may be used to provide evidence to support future public health messages on the optimal duration of sun exposure for cutaneous vitamin D synthesis.

Testing of vitamin D status in Australia was also indicated as a common topic (topic 7, *n* = 40). The first sub-topic 7a (*n* = 14) focused on clinical practice guidelines for testing of vitamin D status. The high number of publications in this topic may be attributed to the change in the Australian Medical Benefits Schedule in 2014 regarding the type of patient that may be reimbursed for testing of vitamin D status (45). The change to the Medical Benefits Schedule was designed to reduce unnecessary vitamin D testing by limiting it to patients at higher risk of vitamin D deficiency, e.g., patients with osteoporosis, osteomalacia, deeply pigmented skin, severe lack of sun exposure, or chronic renal failure (46). However, recent research has shown that such methods to reduce the frequency of vitamin D testing were unsuccessful and may prevent the identification of vitamin D deficiency in individuals that do not fall within those specified categories (45).

Vitamin D assays used in clinical laboratories across Australia are unlikely to be using an assay that has been certified to the reference measurement procedures (RMPs) developed by National Institute of Standards and Technology, Ghent University, and the US Centers for Disease Control and Prevention, which is considered as the ‘gold standard’ for measurement of serum 25(OH)D concentration (47–49). Assays that are not certified to the RMPs may be inaccurate and report lower 25(OH)D concentrations, potentially causing misinterpretation of clinical results if clinicians are unaware (47, 50). Additionally, there is currently no consensus for the optimal level of vitamin D status (51). However, based on the Institute of Medicine (IOM) (52) and the position statement by the Australian and New Zealand Bone and Mineral Society, Osteoporosis Australia, and Endocrine Society of Australia, the recommended serum 25-hydroxyvitamin D concentration for vitamin D sufficiency is ≥50 nmol/L, to support optimal bone health (4).

The other sub-topic, 7b (*n* = 13), focused on testing vitamin D status in Australia with an emphasis on individuals with type 2 diabetes. Vitamin D deficiency may influence pancreatic beta cell function and insulin resistance (53). The associative relationship between vitamin D status and type 2 diabetes has been tested in several cohorts and have identified an inverse association between vitamin D status and type 2 diabetes (54–57). Conversely, causal association between vitamin D and type 2 diabetes have shown inconsistent results in clinical trials, citing small sample sizes, short study duration, low dosage of vitamin D, and the inclusion of subjects with sufficient levels of vitamin D (53).

The current data-driven approach is also useful in identifying potential knowledge gaps in the literature map, highlighting opportunities for further research. There were knowledge gaps that had overlapping discussion points addressed in the earlier sections of the discussion, such as vitamin D status in population groups, health outcomes, and sun exposure. In the following paragraphs, we discuss the knowledge gaps focusing on Aboriginal and Torres Strait Islander peoples and dietary vitamin D.

Despite the higher prevalence of vitamin D deficiency in Aboriginal and Torres Strait Islander peoples, the literature map showed a paucity in the amount of vitamin D literature published (*n* = 9). Although some general Australian population studies may include Aboriginal and Torres Strait Islander peoples in their research (9, 58), they usually only constitute a small percentage of the participants which may not be sufficient to elucidate information specific to Aboriginal and Torres Strait Islander peoples. Hence, population-representative research is needed to provide evidence to support programs and policies to improve vitamin D status in Aboriginal and Torres Strait Islander peoples. Future research guided by Aboriginal and Torres Strait Islander peoples can help to address the limited evidence base and promote vitamin D sufficiency within this population group.

Vitamin D food composition data for Australian retail foods and Australian game products have only recently been published (2, 6). Hence, dietary vitamin D research in Australia is still developing (sub-topic 1cb, *n* = 8; 3 ac, *n* = 8; 4bb, *n* = 8; 4bc, *n* = 8) in comparison to countries such as the United States (59), Canada (60), and Denmark (61), where vitamin D food composition data were developed earlier. The new dietary data in Australia enables further research into dietary vitamin D, which may include the evaluation of diet quality and vitamin D status, investigating socio-economic status and vitamin D status, and estimated population vitamin D intakes from food. The intake of dietary vitamin D in the Aboriginal and Torres Strait Islander peoples in Australia has not yet been estimated. However, recent estimation of dietary intakes of vitamin D from food and beverages in the wider Australian population revealed low dietary intake of vitamin D (<3.5 μg/day) (7). These intake levels are also low in comparison to countries such as the United States (4.9 μg/day) (62), Canada (≤ 5.1 μg/day) (63), Finland (men, 11.2 μg/day; women, 8.6 μg/day) (64), and other European countries (<5 μg/day) (65). Higher vitamin D intakes in Finland may be attributed to the successful implementation of a vitamin D food fortification policy (64).

Through the analysis of excluded publications, we found a paucity of research on VDR and their genomic actions in vitamin D research in Australia. VDRs are found in most human cells, indicating that vitamin D may have a role in various physiological processes beyond musculoskeletal health, such as respiratory health (66, 67), cardiovascular health (68), immune health (69), cancer (14, 70, 71), and neurological conditions (70). However, it is beyond the scope of the literature map to explore the pathophysiological and epidemiological links between vitamin D and these health outcomes; these are discussed more extensively in other reviews (66–71).

The literature map showed vitamin D research trends over time and how that research has been distributed across various research themes in Australia. Each topic follows a hierarchal classification (e.g., health outcomes) with its sub-topic (e.g., MS), which allows the reader to explore their topic of interest from the list of publications for further reading (Supplementary File S1). This approach can be applied globally by researchers who need to synthesise large amounts of literature within a specific field to identify research trends and knowledge gaps.

There were some strengths in this study. Firstly, various types of studies (e.g., observational studies, interventions, and reviews) were included in this literature map. These studies are complementary in providing evidence-based information in vitamin D research. Observational studies are useful in testing associations in large population groups, and interventions are useful in testing potential causative relationships in vitamin D research (72). Hence, the common topics identified in this study provide a good representation of vitamin D research in Australia. Secondly, excluded studies from the initial clustering process were analysed to elucidate areas of emerging research or topics with limited citation links. The excluded studies were also compared against the included studies to assess the variability of research that was excluded.

Our study had some limitations. Although we used strategies such as analysing excluded publications and having a smaller minimum cluster size (*n* = 7) with the aim of providing a comprehensive literature map, there may be pertinent publications that were not identified through this approach. Conducting targeted searches in areas of interest could create a map with higher accuracy for specific topics within vitamin D research. Another limitation of this study was that only one database was used to conduct the literature search: the Web of Science Core Collection database is the only database that has citation data compatibility with the CitNetExplorer software. However, the Web of Science Core Collection database is considered a primary database with high coverage of multidisciplinary research (73). Furthermore, the Web of Science Core Collection database contains the Emerging Sources Citation Index, which includes high-quality peer-reviewed papers. Hence, the publications extracted from the literature search were likely to be comprehensive.

## 5 Conclusion

We provided an overview of vitamin D research in Australia, discussed commonly researched topics, and identified knowledge gaps that could be filled through future research to improve the vitamin D status of Australians. Our literature map revealed that vitamin D deficiency among vulnerable population groups was a prominent research topic in Australia. However, vitamin D research in human health is still a growing field in Australia, and future research may improve knowledge relating to vitamin D in Aboriginal and Torres Strait Islander peoples and dietary vitamin D.

## Data availability statement

The original contributions presented in the study are included in the article/Supplementary material, further inquiries can be directed to the corresponding author.

## Author contributions

BN: Formal analysis, Investigation, Methodology, Project administration, Writing – original draft, Writing – review & editing, Data curation. XQ: Writing – review & editing, Methodology, Software. ED: Supervision, Writing – review & editing. CS: Writing – review & editing, Supervision. EW: Conceptualization, Methodology, Writing – review & editing. NC: Conceptualization, Writing – review & editing, Methodology. LB: Conceptualization, Supervision, Writing – review & editing.
