# Supplementary material for: Mapping the citation network on vitamin D research in Australia: a data-driven approach
Source: Front Med (Lausanne). 2023 Nov 28;10:1298190. doi: 10.3389/fmed.2023.1298190 (PMC10713817; doi:10.3389/fmed.2023.1298190)
Supplement: Supplementary file 1 [file Presentation_1.pdf]

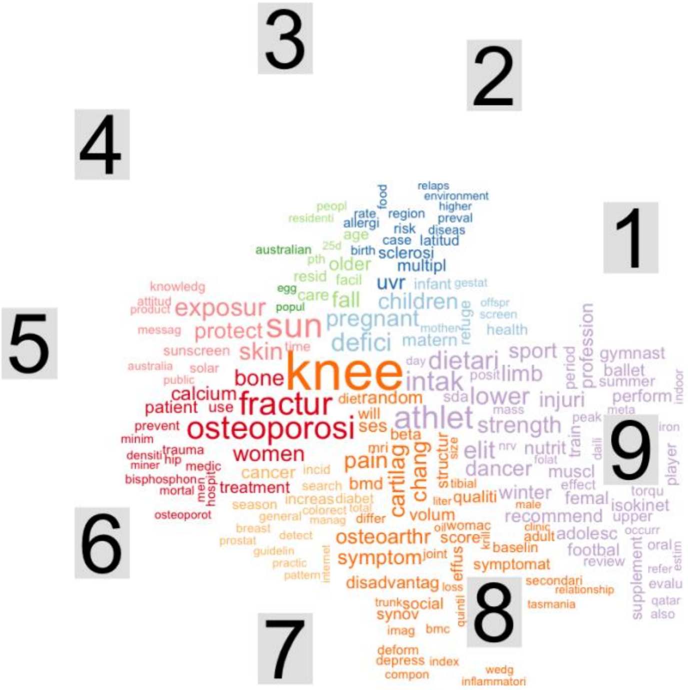

Supplementary Figure S1. A comparison cloud of the top 200 most unique words in cluster 1 to 9. Light blue, cluster 1; dark blue, cluster 2; light green, cluster 3; dark green, cluster 4; pink, cluster 5; red, cluster 6; light orange, cluster 7; orange, cluster 8; purple, cluster 9.

Supplementary Table S1. Topic 1:140 publications, Vitamin D in vulnerable populations and its impact on child development

Citation network	Word cloud
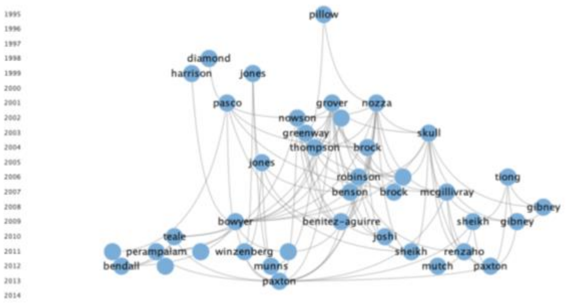	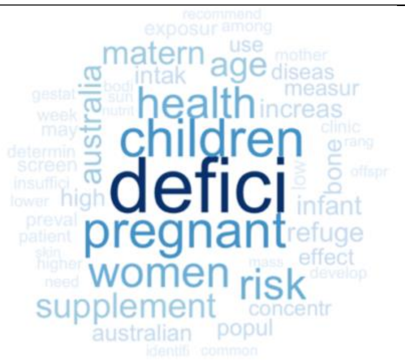

Sub-topics and number of publications	Indicative publication	Word cloud	Top 5 most unique topic terms
1a: (n=44) Vitamin D deficiency in Australians and migrants	Nowson CA, Margerison C. Vitamin D intake and vitamin D status of Australians. Med J Aust. 2002;177(3):149-152. doi: 10.5694/j.1326-5377.2002.tb04702.x. (1)	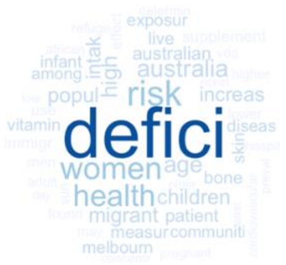	defici*<br>risk<br>high<br>migrant*<br>popul*
1aa: (n=27) Vitamin D deficiency in women and child migrants	Grover SR, Morley R. Vitamin D deficiency in veiled or dark-skinned pregnant women. Med J Aust. 2001;175(5):251-252. doi: 10.5694/j.1326-5377.2001.tb143558.x. (2)	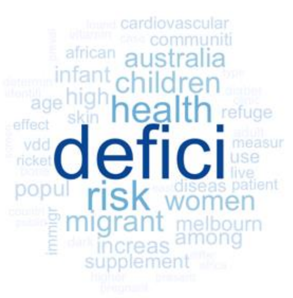	defici*<br>risk<br>health<br>children<br>migrant*
1aaa: (n=16) Vitamin D deficiency in adult migrant women	Skull SA, Ngeow JY, Biggs BA, Street A, Ebeling PR. Vitamin D deficiency is common and unrecognized among recently arrived adult immigrants from The Horn of Africa. Intern Med J. 2003;33(1-2):47-51. doi: 10.1046/j.1445-5994.2003.00344.x. (3)	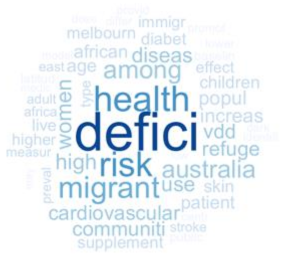	health<br>risk<br>migrant*<br>among<br>Australia

1aab: (n=11) Vitamin D deficiency in children and infants	Nozza JM, Rodda CP. Vitamin D deficiency in mothers of infants with rickets. Med J Aust. 2001;175(5):253-255. doi: 10.5694/j.1326-5377.2001.tb143559.x. (4)	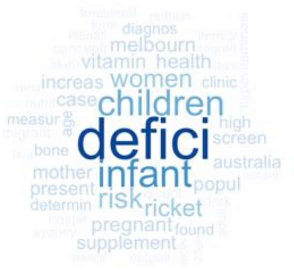	defici* infant children women ricket
1ab: (n=9) Testing for Vitamin D deficiency in older adults	Joshi D, Center JR, Eisman JA. Vitamin D deficiency in adults. Aust Prescr. 2010;33:103-106. doi:10.18773/austprescr.2010.053. (5)	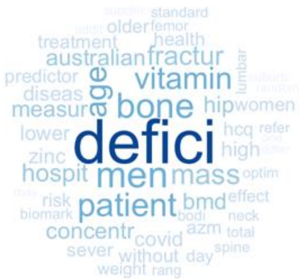	men bone patient age vitamin
1ac: (n=8) Vitamin D deficiency in Australian women	Brock K, Wilkinson M, Cook R, Lee S, Bermingham M. Associations with Vitamin D deficiency in "at risk" Australians. J Steroid Biochem Mol Biol. 2004;89-90(1-5):581-588. doi: 10.1016/j.jsbmb.2004.03.073. (6)	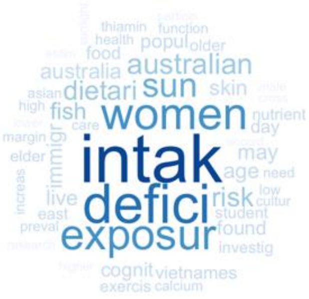	intak* women exposur* sun Australian
1b: (n=43) Vitamin D deficiency in pregnant women	Paxton GA, Teale GR, Nowson CA, Mason RS, McGrath JJ, Thompson MJ, Siafarikas A, Rodda CP, Munns CF; Australian and New Zealand Bone and Mineral Society; Osteoporosis Australia. Vitamin D and health in pregnancy, infants, children and adolescents in Australia and New Zealand: a position statement. Med J Aust. 2013;198(3):142-143. doi: 10.5694/mja11.11592. (7)	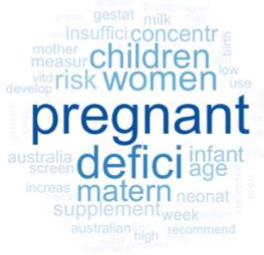	pregnant women matern* age concentr*
	1ba: (n=18) Vitamin D deficiency in pregnancy	Bowyer L, Catling-Paull C, Diamond T, Homer C, Davis G, Craig ME. Vitamin D, PTH and calcium levels in pregnant women and their neonates. Clin Endocrinol (Oxf). 2009;70(3):372-377. doi:	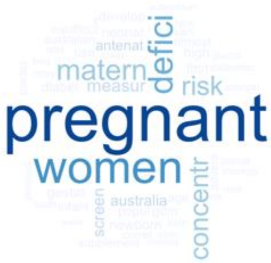	pregnant women defici* concentr* risk

	10.1111/j.1365-2265.2008.03316.x. (8)		
1baa: (n=11) Testing for vitamin D status in pregnant women	Teale GR, Cunningham CE. Vitamin D deficiency is common among pregnant women in rural Victoria. Aust N Z J Obstet Gynaecol. 2010;50(3):259-261. doi: 10.1111/j.1479-828X.2010.01147.x. (9)	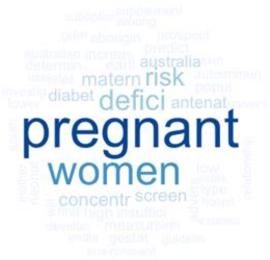	pregnant women defici* risk screen*
1bab: (n=7) Association between maternal and newborn vitamin D status	Thomas SD, Fudge AN, Whiting M, Coates PS. The correlation between third-trimester maternal and newborn-serum 25-hydroxy-vitamin D in a selected South Australian group of newborn samples. BMJ Open. 2011;1(2):e000236. doi: 10.1136/bmjopen-2011-000236. (10)	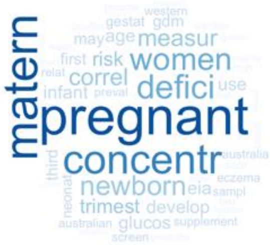	matern* concentr* newborn measur* correl*
1bb: (n=15) Vitamin D deficiency and milk intake in children	Munns CF, Simm PJ, Rodda CP, Garnett SP, Zacharin MR, Ward LM, Geddes J, Cherian S, Zurynski Y, Cowell CT; APSU Vitamin D Study Group. Incidence of vitamin D deficiency rickets among Australian children: an Australian Paediatric Surveillance Unit study. Med J Aust. 2012;196(7):466-468. doi: 10.5694/mja11.10662. (11)	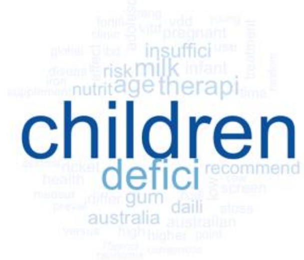	children therapi* milk insuffici* gum (Growing up milk)
1bba: (n=8) Vitamin D status and growing up milk for children	Matsuyama M, Harb T, David M, Davies PS, Hill RJ. Effect of fortified milk on growth and nutritional status in young children: a systematic review and meta-analysis. Public Health Nutr. 2017;20(7):1214-1225. doi: 10.1017/S1368980016003189. (12)	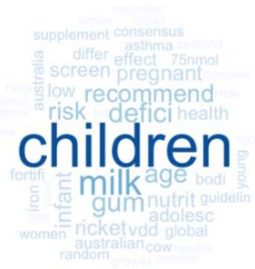	milk gum (Growing up milk) age recommend risk

1bbb: (n=7) Treatment for children with vitamin D deficiency	Rigterink T, Appleton L, Day AS. Vitamin D therapy in children with inflammatory bowel disease: A systematic review. World J Clin Pediatr. 2019;8(1):1-14. doi: 10.5409/wjcp.v8.i1.1. (13)	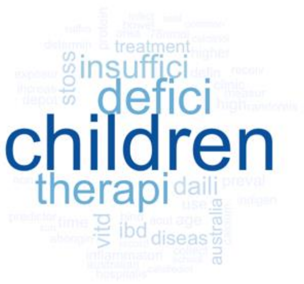	children<br>defici*<br>therapi*<br>insuffici*<br>daili*
1bc: (n=7) Maternal vitamin D and child development	Whitehouse AJ, Holt BJ, Serralha M, Holt PG, Kusel MM, Hart PH. Maternal serum vitamin D levels during pregnancy and offspring neurocognitive development. Pediatrics. 2012;129(3):485-493. doi: 10.1542/peds.2011-2644. (14)	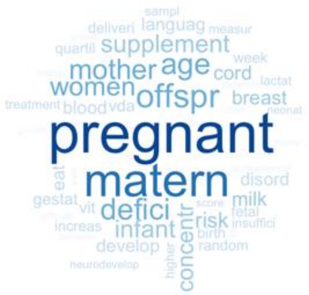	matern*<br>offspr*<br>age<br>mother<br>infant
1c: (n=26) Vitamin D deficiency and bone health in children	Pasco JA, Henry MJ, Nicholson GC, Sanders KM, Kotowicz MA. Vitamin D status of women in the Geelong Osteoporosis Study: association with diet and casual exposure to sunlight. Med J Aust. 2001;175(8):401-405. doi: 10.5694/j.1326-5377.2001.tb143643.x. (15)	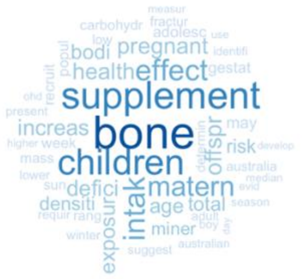	bone<br>supplemen<br>t<br>intak*<br>effect<br>offspr*
1ca: (n=12) Vitamin D supplement intake and bone health in children	Jones G, Dwyer T, Hynes KL, Parameswaran V, Greenaway TM. Vitamin D insufficiency in adolescent males in Southern Tasmania: prevalence, determinants, and relationship to bone turnover markers. Osteoporos Int. 2005;16(6):636-641. doi: 10.1007/s00198-004-1733-z. (16)	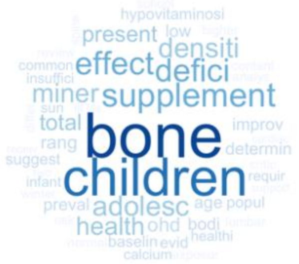	bone<br>children<br>supplemen<br>t<br>defici*<br>effect
1cb: (n=8) Vitamin D intake during gestation and bone health of children	Pasco JA, Wark JD, Carlin JB, Ponsonby AL, Vuillermin PJ, Morley R. Maternal vitamin D in pregnancy may influence not only offspring bone mass but other aspects of musculoskeletal health and adiposity. Med Hypotheses. 2008;71(2):266-269. doi:	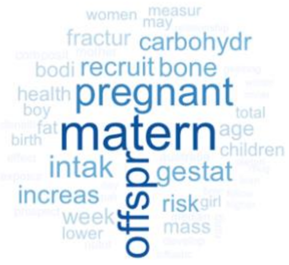	matern*<br>offspr*<br>pregnanc*<br>intak*<br>gestation

	10.1016/j.mehy.2008.01.033. (17)		
1d: (n=17) Vitamin D deficiency in child refugees	Tiong AC, Patel MS, Gardiner J, Ryan R, Linton KS, Walker KA, Scopel J, Biggs BA. Health issues in newly arrived African refugees attending general practice clinics in Melbourne. Med J Aust. 2006;185(11-12):602-606. doi: 10.5694/j.1326-5377.2006.tb00724.x. (18)	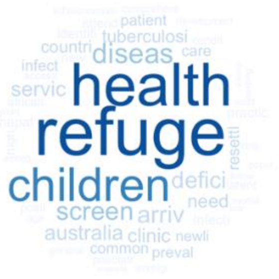	refuge* health children diseas* screen*

Note: The citation network provides an overview of the top 40 citation links within the topic. The nodes indicate the first author of the publication, and the lines linking the nodes represents the citation links. The publication year is plotted on the left. The word cloud shows the frequency words within the topic and sub-topics, where words with a higher frequency have a larger size and opacity.

Supplementary Table S2. Topic 2: 116 publications, Impact of sun exposure and UVB radiation on various health conditions

Citation network	Word cloud
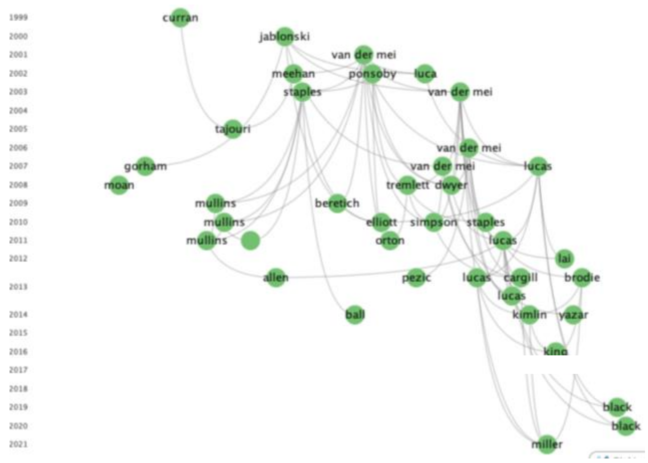	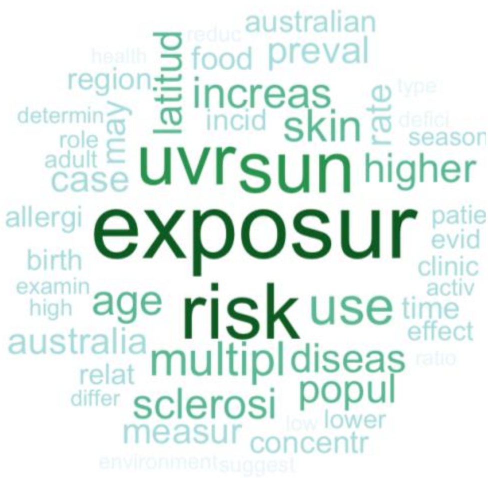

Sub-topics and number of publications	Indicative publication	Word cloud	Top 5 most unique topic terms
2a: (n=34) Vitamin D and multiple sclerosis	van der Mei IA, Ponsonby AL, Dwyer T, Blizzard L, Taylor BV, Kilpatrick T, Butzkueven H, McMichael AJ. Vitamin D levels in people with multiple sclerosis and community controls in Tasmania, Australia. J Neurol. 2007;254(5):581-590. doi: 10.1007/s00415-006-0315-8. (19)	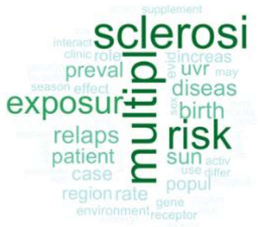	multipl* sclerosi* risk relaps* birth
2b: (n=34) Impact of the environment on vitamin D status and CNS demyelination	Lucas RM, Ponsonby AL, Dear K, Valery PC, Pender MP, Taylor BV, Kilpatrick TJ, Dwyer T, Coulthard A, Chapman C, van der Mei I, Williams D, McMichael AJ. Sun exposure and vitamin D are independent risk factors for CNS demyelination. Neurology. 2011;76(6):540-548. doi: 10.1212/WNL.0b013e31820af93d. (20)	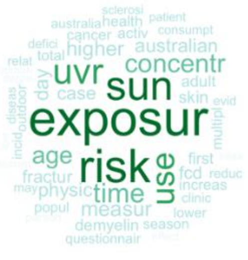	time day Australian physic* fractur*

2ba: (n=16) Latitude gradient and vitamin D status	Lucas RM, Ponsonby AL, Dear K, Valery PC, Taylor B, van der Mei I, McMichael AJ, Pender MP, Chapman C, Coulthard A, Kilpatrick TJ, Stankovich J, Williams D, Dwyer T. Vitamin D status: multifactorial contribution of environment, genes and other factors in healthy Australian adults across a latitude gradient. J Steroid Biochem Mol Biol. 2013;136:300-308. doi: 10.1016/j.jsbmb.2013.01.011. (21)	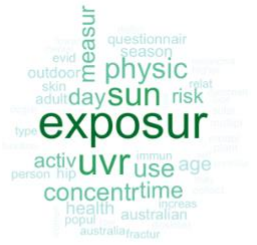	exposur*<br>uvr<br>sun<br>physic*<br>use
2bb: (n=12) Vitamin D status and CNS demyelination	Lucas R, Ponsonby AL, McMichael A, van der Mei I, Chapman C, Coulthard A, Dear K, Dwyer T, Kilpatrick T, Pender M, Taylor B, Valery P, Williams D. Observational analytic studies in multiple sclerosis: controlling bias through study design and conduct. The Australian Multicentre Study of Environment and Immune Function. Mult Scler. 2007;13(7):827-839. doi: 10.1177/1352458507077174. (22)	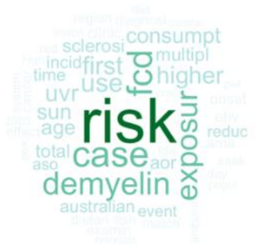	risk<br>case<br>fcd (CNS demyelination)<br>demyelin*<br>first
2c: (n=19) Vitamin D and food allergies	Staples JA, Ponsonby AL, Lim LL, McMichael AJ. Ecologic analysis of some immune-related disorders, including type 1 diabetes, in Australia: latitude, regional ultraviolet radiation, and disease prevalence. Environ Health Perspect. 2003;111(4):518-523. doi: 10.1289/ehp.5941. (23)	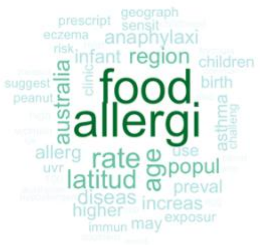	food<br>allergi*<br>rate<br>age<br>region

2d: (n=13) Vitamin D status and melanoma	Jablonski NG, Chaplin G. The evolution of human skin coloration. J Hum Evol. 2000;39(1):57-106. doi: 10.1006/jhev.2000.0403. (24)	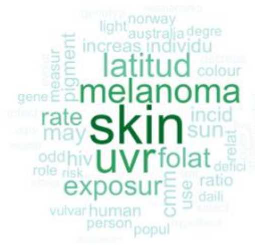	skin melanoma folat* may cmm (cutaneous malignant melanoma)
2e: (n=9) Vitamin D status and myopia risk	van der Mei IA, Blizzard L, Ponsonby AL, Dwyer T. Validity and reliability of adult recall of past sun exposure in a case-control study of multiple sclerosis. Cancer Epidemiol Biomarkers Prev. 2006;15(8):1538-1544. doi: 10.1158/1055-9965.EPI-05-0969. (25)	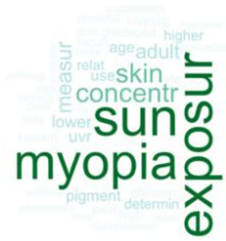	sun exposur* myopia concentr* measur*
2f: (n=7) Vitamin D status and type 1 diabetes	Elliott JC, Lucas RM, Clements MS, Bambrick HJ. Population density determines the direction of the association between ambient ultraviolet radiation and type 1 diabetes incidence. Pediatr Diabetes. 2010;11(6):394-402. doi: 10.1111/j.1399-5448.2009.00620.x. (26)	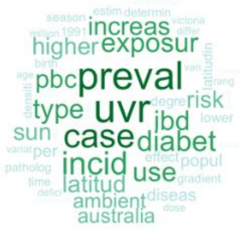	uvr preval* case incid* diabet*

Note: The citation network provides an overview of the top 40 citation links within the topic. Each node is labelled with the surname of the first author of the citation, and the lines linking the nodes represents the citation links. The publication year is plotted on the left. The word cloud shows the frequency words within the topic and sub-topics, where words with a higher frequency have a larger size and opacity.

Supplementary Table S3. Topic 3: 115 publications, Vitamin D and falls and fractures in older adults.

Citation network	Word cloud
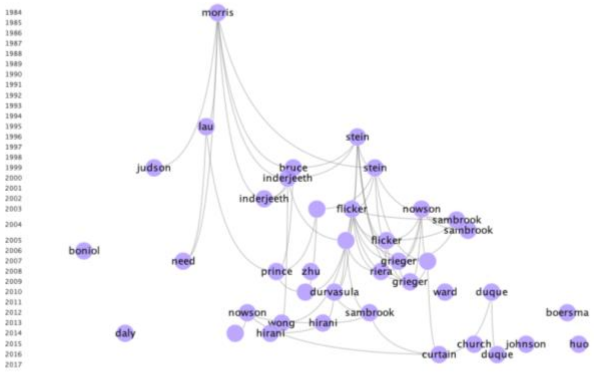	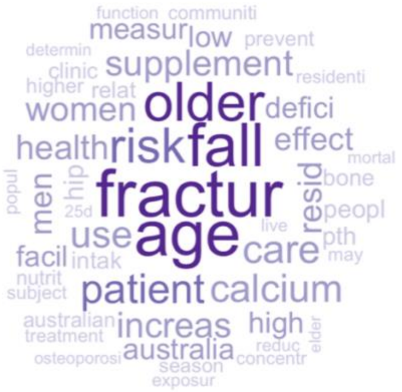

Sub-topics and number of publications	Indicative publication	Word cloud	Top 5 most unique topic terms
3a: (n=29) Vitamin D deficiency and fractures in RACFs	Flicker L, Mead K, MacInnis RJ, Nowson C, Scherer S, Stein MS, Thomasx J, Hopper JL, Wark JD. Serum vitamin D and falls in older women in residential care in Australia. J Am Geriatr Soc. 2003;51(11):1533-1538. doi: 10.1046/j.1532-5415.2003.51510.x. (27)	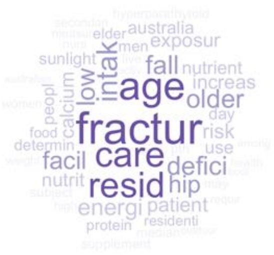	age<br>intak*<br>defici*<br>low<br>hip
3aa: (n=10) Hyperparathyroidism and fractures in RACFs	Stein MS, Scherer SC, Walton SL, Gilbert RE, Ebeling PR, Flicker L, Wark JD. Risk factors for secondary hyperparathyroidism in a nursing home population. Clin Endocrinol (Oxf). 1996;44(4):375-383. doi: 10.1046/j.1365-2265.1996.701521.x. (28)	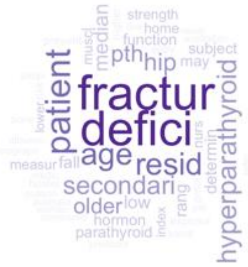	defici*<br>fractur*<br>patient<br>resid*<br>hyperparathyroid

3ab: (n=9) Sun exposure and hip fracture risk in RACFs	Sambrook PN, Cameron ID, Chen JS, Cumming RG, Durvasula S, Herrmann M, Kok C, Lord SR, Macara M, March LM, Mason RS, Seibel MJ, Wilson N, Simpson JM. Does increased sunlight exposure work as a strategy to improve vitamin D status in the elderly: a cluster randomised controlled trial. Osteoporos Int. 2012;23(2):615-624. doi: 10.1007/s00198-011-1590-5. (29)	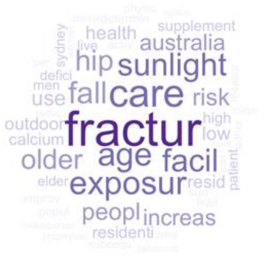	care<br>age<br>exposur*<br>sunlight<br>facil*
3ac: (n=8) Vitamin D intake in RACFs	Nowson CA, Sherwin AJ, McPhee JG, Wark JD, Flicker L. Energy, protein, calcium, vitamin D and fibre intakes from meals in residential care establishments in Australia. Asia Pac J Clin Nutr. 2003;12(2):172-177. (30)	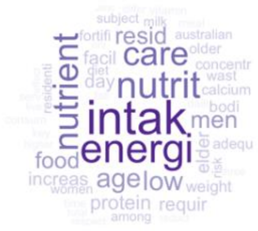	intak*<br>energi*<br>nutrit*<br>nutrient<br>low
3b: (n=18) Vitamin D status and hospitalised patients	Quaggiotto P, Tran H, Bhanugopan M. Vitamin D deficiency remains prevalent despite increased laboratory testing in New South Wales, Australia. Singapore Med J. 2014;55(5):271-280. doi: 10.11622/smedj.2014071. (31)	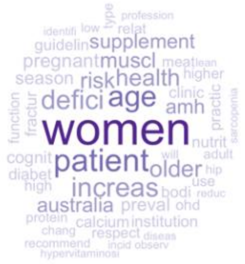	women<br>patient<br>amh<br>pregnant<br>preval*

3c: (n=15) Vitamin D supplement intake in RACFs	Flicker L, MacInnis RJ, Stein MS, Scherer SC, Mead KE, Nowson CA, Thomas J, Lowndes C, Hopper JL, Wark JD. Should older people in residential care receive vitamin D to prevent falls? Results of a randomized trial. J Am Geriatr Soc. 2005;53(11):1881-1888. doi: 10.1111/j.1532-5415.2005.00468.x. (32)	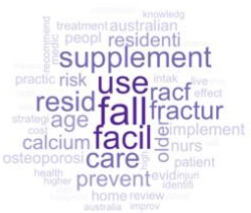	facil*<br>use<br>supplement<br>care<br>resid*
3d: (n=13) Serum parathyroid hormone and fall risk in older adults	Sambrook PN, Chen JS, March LM, Cameron ID, Cumming RG, Lord SR, Zochling J, Sitoh YY, Lau TC, Schwarz J, Seibel MJ. Serum parathyroid hormone predicts time to fall independent of vitamin D status in a frail elderly population. J Clin Endocrinol Metab. 2004;89(4):1572-1576. doi: 10.1210/jc.2003-031782. (33)	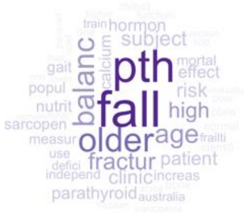	fall<br>pth<br>older<br>balanc*<br>high
3e: (n=13) Vitamin D status and aging in men	Hirani V, Naganathan V, Cumming RG, Blyth F, Le Couteur DG, Handelsman DJ, Waite LM, Seibel MJ. Associations between frailty and serum 25-hydroxyvitamin D and 1,25-dihydroxyvitamin D concentrations in older Australian men: the Concord Health and Ageing in Men Project. J Gerontol A Biol Sci Med Sci. 2013;68(9):1112-1121. doi:	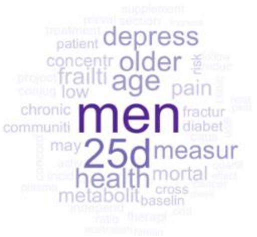	men<br>25d<br>health<br>depress*<br>measur*

	10.1093/gerona/glt059. (34)		
3f: (n=13) Association between low vitamin D status and falls and fracture risk in older adults	Latham NK, Anderson CS, Lee A, Bennett DA, Moseley A, Cameron ID; Fitness Collaborative Group. A randomized, controlled trial of quadriceps resistance exercise and vitamin D in frail older people: the Frailty Interventions Trial in Elderly Subjects (FITNESS). J Am Geriatr Soc. 2003;51(3):291-299. doi: 10.1046/j.1532-5415.2003.51101.x (35)	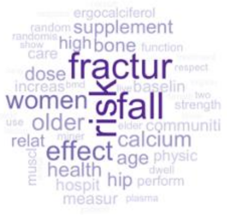	risk<br>fractur*<br>effect<br>dose<br>relat*
3g: (n=10) Vitamin D status and bone mineral density	Morris HA, Morrison GW, Burr M, Thomas DW, Nordin BE. Vitamin D and femoral neck fractures in elderly South Australian women. Med J Aust. 1984;140(9):519-521. doi: 10.5694/j.1326-5377.1984.tb108222.x. (36)	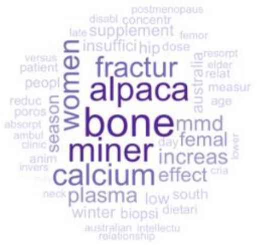	bone<br>miner*<br>alpaca<br>calcium<br>increas*

Note: The citation network provides an overview of the top 40 citation links within the topic. Each node is labelled with the surname of the first author of the citation, and the lines linking the nodes represents the citation links. The publication year is plotted on the left. The word cloud shows the frequency words within the topic and sub-topics, where words with a higher frequency have a larger size and opacity.

Supplementary Table S4. Topic 4: 110 publications, Vitamin D and its association with health outcomes

Citation network	Word cloud
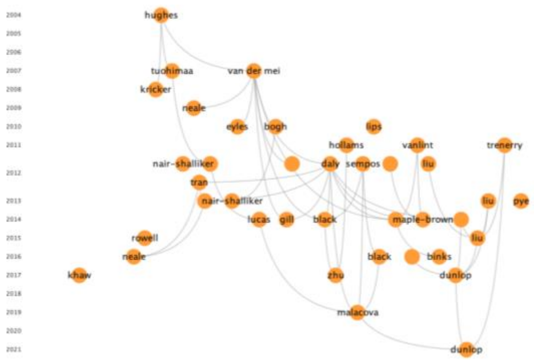	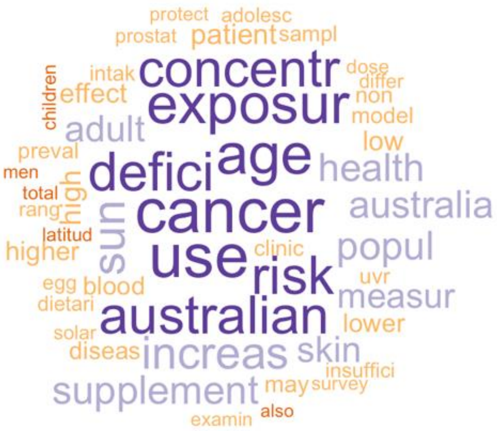

Sub-topics and number of publications	Indicative publication	Word cloud	Top 5 most unique topic terms
4a: (n=29) Vitamin D supplementation and health outcomes	Daly RM, Gagnon C, Lu ZX, Magliano DJ, Dunstan DW, Sikaris KA, Zimmet PZ, Ebeling PR, Shaw JE. Prevalence of vitamin D deficiency and its determinants in Australian adults aged 25 years and older: a national, population-based study. Clin Endocrinol (Oxf). 2012;77(1):26-35. doi: 10.1111/j.1365-2265.2011.04320.x. (37)	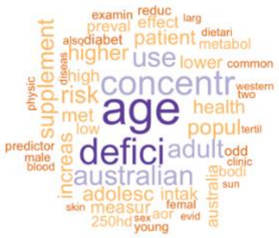	age<br>concentr*<br>adult<br>supplemen t<br>higher
4aa: (n=13) Vitamin D deficiency and metabolic syndrome	Gagnon C, Lu ZX, Magliano DJ, Dunstan DW, Shaw JE, Zimmet PZ, Sikaris K, Ebeling PR, Daly RM. Low serum 25-hydroxyvitamin D is associated with increased risk of the development of the metabolic syndrome at five years: results from a national, population-based prospective study (The Australian Diabetes, Obesity and Lifestyle Study: AusDiab). J	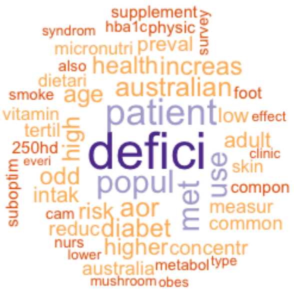	defici*<br>patient<br>popul*<br>met<br>aor*

	Clin Endocrinol Metab. 2012;97(6):1953-1961. doi: 10.1210/jc.2011-3187. (38)		
4ab: (n=9) Vitamin D status and health outcomes of Australian adolescents	Black LJ, Burrows SA, Jacoby P, Oddy WH, Beilin LJ, Chan She Ping-Delfos W, Marshall CE, Holt PG, Hart PH, Mori TA. Vitamin D status and predictors of serum 25-hydroxyvitamin D concentrations in Western Australian adolescents. Br J Nutr. 2014;112(7):1154-1162. doi: 10.1017/S000711451400186X. (39)	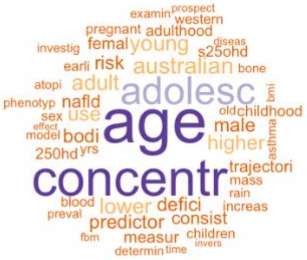	age concentr* adolesc* Australian adult
4b: (n=27) Vitamin D deficiency in the Australian population	Malacova E, Cheang PR, Dunlop E, Sherriff JL, Lucas RM, Daly RM, Nowson CA, Black LJ. Prevalence and predictors of vitamin D deficiency in a nationally representative sample of adults participating in the 2011-2013 Australian Health Survey. Br J Nutr. 2019;121(8):894-904. doi: 10.1017/S0007114519000151. (40)	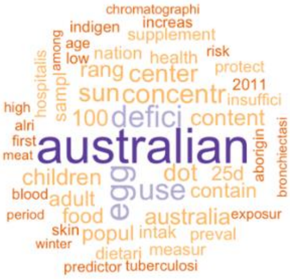	Australian egg center dot Australia
4ba: (n=9) Vitamin D deficiency in Aboriginal and Torres Strait Islander peoples	Vanlint SJ, Morris HA, Newbury JW, Crockett AJ. Vitamin D insufficiency in Aboriginal Australians. Med J Aust. 2011;194(3):131-4. doi: 10.5694/j.1326-5377.2011.tb04195.x. (41)	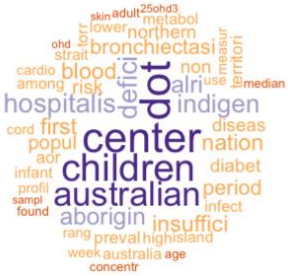	center dot children defici* hospitalis*
4bb: (n=8) Vitamin D content in Australian foods	Dunlop E, Cunningham J, Sherriff JL, Lucas RM, Greenfield H, Arcot J, Strobel N, Black LJ. Vitamin D_3 and 25-Hydroxyvitamin D_3 Content of Retail White Fish and Eggs in Australia. Nutrients. 2017;9(7):647. doi: 10.3390/nu9070647. (42)	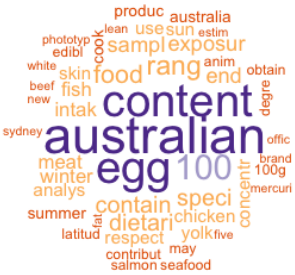	Australian content egg 100 rang*

4bc: (n=8) Vitamin D status and vitamin D intake from food and supplements	Black LJ, Jacoby P, Nowson CA, Daly RM, Lucas RM. Predictors of Vitamin D-Containing Supplement Use in the Australian Population and Associations between Dose and Serum 25-Hydroxyvitamin D Concentrations. Nutrients. 2016;8(6):356. doi: 10.3390/nu8060356. (43)	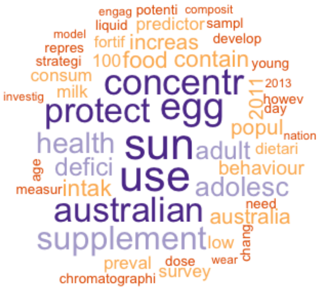	sun use concentr* protect supplement
4c: (n=16) Sun exposure and cancer risk	Hughes AM, Armstrong BK, Vajdic CM, Turner J, Grulich AE, Fritschi L, Milliken S, Kaldor J, Benke G, Kricker A. Sun exposure may protect against non-Hodgkin lymphoma: a case-control study. Int J Cancer. 2004;112(5):865-871. doi: 10.1002/ijc.20470. (44)	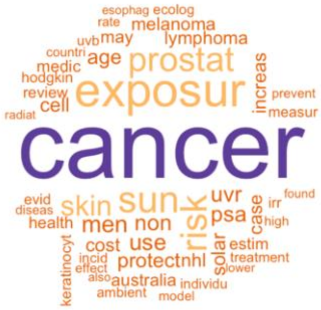	cancer sun risk prostat* skin
4d: (n=16) Vitamin D deficiency and the lifecycle	van der Mei IA, Ponsonby AL, Engelsen O, Pasco JA, McGrath JJ, Eyles DW, Blizzard L, Dwyer T, Lucas R, Jones G. The high prevalence of vitamin D insufficiency across Australian populations is only partly explained by season and latitude. Environ Health Perspect. 2007;115(8):1132-1139. doi: 10.1289/ehp.9937. (45)	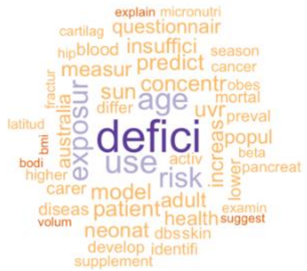	defici* use model patient predict*
4e: (n=13) Vitamin D status and UVB radiation from sun exposure	Bogh MK, Schmedes AV, Philipsen PA, Thieden E, Wulf HC. Vitamin D production after UVB exposure depends on baseline vitamin D and total cholesterol but not on skin pigmentation. J Invest Dermatol. 2010;130(2):546-553. doi: 10.1038/jid.2009.323. (46)	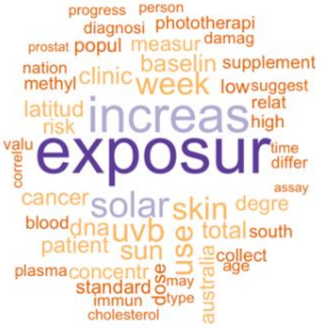	exposur* increase* solar uvb week

Note: The citation network provides an overview of the top 40 citation links within the topic. Each node is labelled with the surname of the first author of the citation, and the lines linking the nodes represents the citation links. The publication year is plotted on the left. The word cloud shows the frequency words within the topic and sub-topics, where words with a higher frequency have a larger size and opacity.

Supplementary Table S5. Topic 5: 71 publications, Vitamin D from sun exposure

<table>
<tr><th>Citation network</th><th>Word cloud</th></tr>
<tr><td>
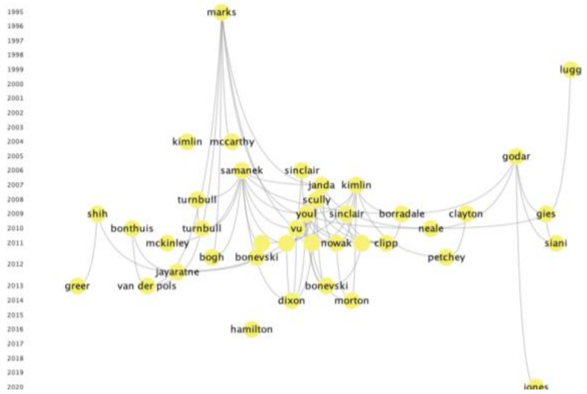

</td><td>
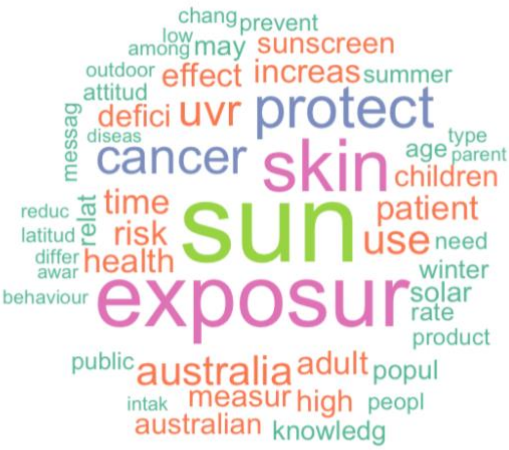

</td></tr>
</table>

<table>
<tr><th>Sub-topics and number of publications</th><th>Indicative publication</th><th>Word cloud</th><th>Top 5 most unique topic terms</th></tr>
<tr><td>5a: (n=20) Knowledge and attitudes of Australians towards sun protection</td><td>Youl PH, Janda M, Kimlin M. Vitamin D and sun protection: the impact of mixed public health messages in Australia. Int J Cancer. 2009;124(8):1963-1970. doi: 10.1002/ijc.24154. (47)</td><td>
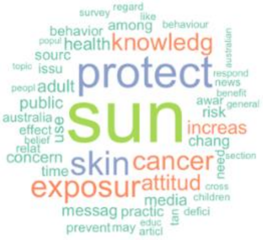

</td><td>sun
protect
skin
cancer
knowledge</td></tr>
<tr><td>5b: (n=18) Vitamin D status and sunscreen use in Australia</td><td>Marks R, Foley PA, Jolley D, Knight KR, Harrison J, Thompson SC. The effect of regular sunscreen use on vitamin D levels in an Australian population. Results of a randomized controlled trial. Arch Dermatol. 1995;131(4):415-421. doi: 10.1001/archderm.1995. 01690160043006. (48)</td><td>
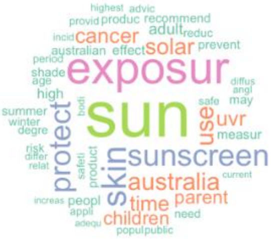

</td><td>sunscreen
use
Australia
solar
time</td></tr>
<tr><td>5c: (n=12) Vitamin D status and seasons in Australia</td><td>Kimlin M, Harrison S, Nowak M, Moore M, Brodie A, Lang C. Does a high UV environment ensure adequate vitamin D status?
J Photochem Photobiol B. 2007;89(2-3):139-147. doi: 10.1016/j.jphotobiol.2007.09.00 8. (49)</td><td>
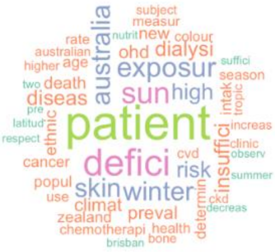

</td><td>patient
defici*
winter
high
risk</td></tr>
</table>

5d: (n=10) Lower vitamin D status with limited sun exposure	Jayaratne N, Russell A, van der Pols JC. Sun protection and vitamin D status in an Australian subtropical community. Prev Med. 2012;55(2):146-150. doi: 10.1016/j.ypmed.2012.05.011. (50)	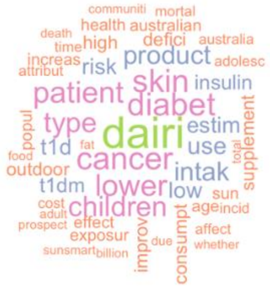	dairi* diabet* lower children type
5e: (n=8) Measuremen t of UVB radiation exposure in Australia	Godar DE. UV doses worldwide. Photochem Photobiol. 2005;81(4):736-749. doi: 10.1562/2004-09-07-ir-308r.1. (51)	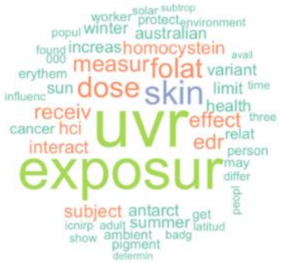	uvr exposur* dose folat* measur*

Note: The citation network provides an overview of the top 40 citation links within the topic. Each node is labelled with the surname of the first author of the citation, and the lines linking the nodes represents the citation links. The publication year is plotted on the left. The word cloud shows the frequency words within the topic and sub-topics, where words with a higher frequency have a larger size and opacity.

Supplementary Table S6. Topic 6: 62 publications, Vitamin D, calcium, and musculoskeletal health

Citation network	Word cloud
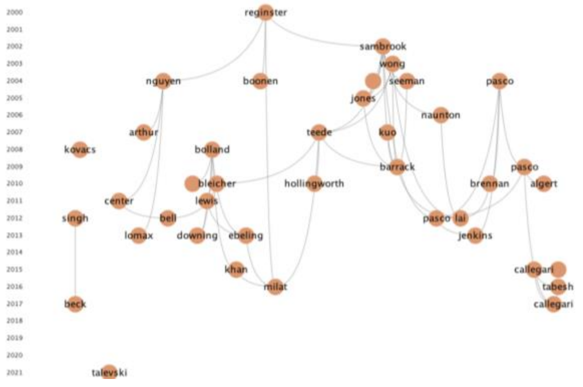	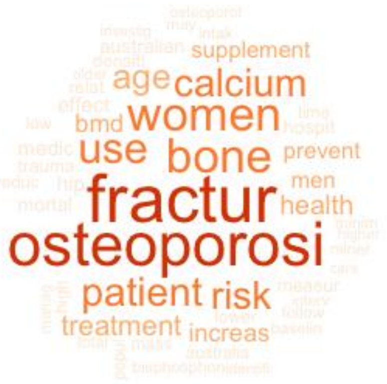

Sub-topics and number of publications	Indicative publication	Word cloud	Most unique topic terms
6a: (n=17) Vitamin D and bone health in women	Pasco JA, Henry MJ, Kotowicz MA, Sanders KM, Seeman E, Pasco JR, Schneider HG, Nicholson GC. Seasonal periodicity of serum vitamin D and parathyroid hormone, bone resorption, and fractures: the Geelong Osteoporosis Study. J Bone Miner Res. 2004;19(5):752-758. doi: 10.1359/JBMR.040125. (52)	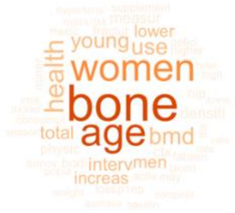	bone<br>age<br>women<br>health<br>bmd (bone mineral density)
6aa: (n=10) Association between age, vitamin D and bone mineral density	Pasco JA, Henry MJ, Nicholson GC, Brennan SL, Kotowicz MA. Behavioural and physical characteristics associated with vitamin D status in women. Bone. 2009;44(6):1085-1091. doi: 10.1016/j.bone.2009.02.020. (53)	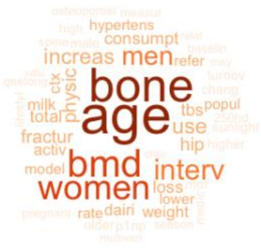	age<br>bmd (bone mineral density)<br>interv*<br>men<br>increas*
6ab: (n=7) Vitamin D status and bone mineral density in	Callegari ET, Reavley N, Garland SM, Gorelik A, Wark JD. Vitamin D Status, Bone Mineral Density and Mental Health in Young Australian Women: The Safe-D Study. J Public	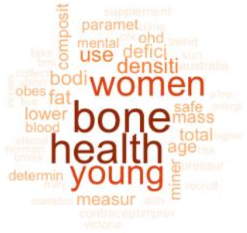	bone<br>health<br>women<br>young<br>densiti*

young women	Health Res. 2015;4(3):594. doi: 10.4081/jphr.2015.594. (54)		
6b: (n=15) Prevention and treatment of osteoporosis in patients with minimal trauma fracture	Sambrook PN, Seeman E, Phillips SR, Ebeling PR; Osteoporosis Australia; National Prescribing Service. Preventing osteoporosis: outcomes of the Australian Fracture Prevention Summit. Med J Aust. 2002;176(S8):S1-16. doi: 10.5694/j.1326-5377.2002.tb04475.x. (55)	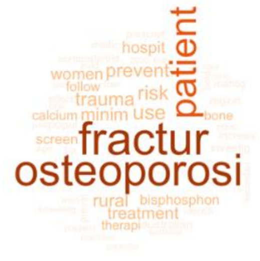	osteoporosi* patient prevent trauma minim*
6c: (n=13) Calcium supplement and bone health	Ebeling PR, Daly RM, Kerr DA, Kimlin MG. Building healthy bones throughout life: an evidence-informed strategy to prevent osteoporosis in Australia. Med J Aust. 2013;199(S7):S1-S46. doi: 10.5694/j.1326-5377.2013.tb04225.x. (56)	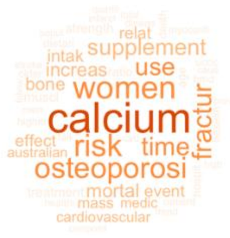	calcium risk time supplement increas*
6d: (n=9) Preventing osteoporotic fractures with vitamin D and calcium supplement	Nguyen TV, Center JR, Eisman JA. Osteoporosis: underrated, underdiagnosed and undertreated. Med J Aust. 2004;180(S5):S18-22. doi: 10.5694/j.1326-5377.2004.tb05908.x. (57)	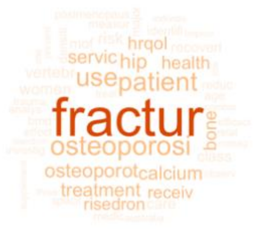	fractur* use hip osteoporot* servic*
6e: (n=8) Treatment of osteoporosis	Bleicher K, Naganathan V, Cumming RG, Seibel MJ, Sambrook PN, Blyth FM, Le Couteur DG, Handelsman DJ, Waite LM, Creasey HM. Prevalence and treatment of osteoporosis in older Australian men: findings from the CHAMP study. Med J Aust. 2010;193(7):387-391. doi: 10.5694/j.1326-5377.2010.tb03964.x. (58)	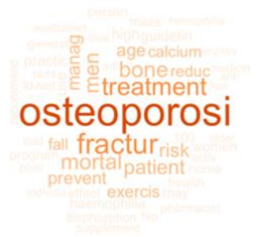	treatment mortal* men manag* exercis*

Note: The citation network provides an overview of the top 40 citation links within the topic. Each node is labelled with the surname of the first author of the citation, and the lines linking

the nodes represents the citation links. The publication year is plotted on the left. The word cloud shows the frequency words within the topic and sub-topics, where words with a higher frequency have a larger size and opacity.

Supplementary Table S7. Topic 7: 40 publications, Testing of vitamin D status in Australia

Citation network	Word cloud
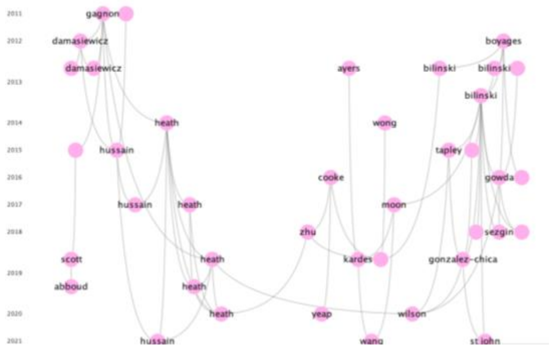	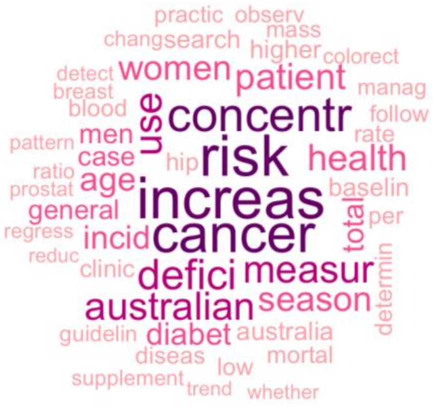

Sub-topics and number of publications	Indicative publication	Word cloud	Most unique topic terms
7a: (n=14) Clinical practice guidelines for vitamin D testing	Bilinski K, Boyages S Evidence of overtesting for vitamin D in Australia: an analysis of 4.5 years of Medicare Benefits Schedule (MBS) data. BMJ Open. 2013; 3:e002955. doi: 10.1136/bmjopen-2013-002955. (59)	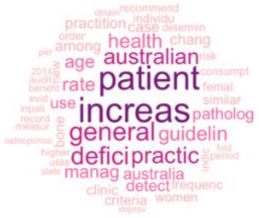	increas*<br>patient<br>general<br>defici*<br>practic*
7b: (n=13) Testing Vitamin D status and incidence of type 2 diabetes	Gagnon C, Lu ZX, Magliano DJ, Dunstan DW, Shaw JE, Zimmet PZ, Sikaris K, Grantham N, Ebeling PR, Daly RM. Serum 25-hydroxyvitamin D, calcium intake, and risk of type 2 diabetes after 5 years: results from a national, population-based prospective study (the Australian Diabetes, Obesity and Lifestyle study). Diabetes Care. 2011;34(5):1133-1138. doi:	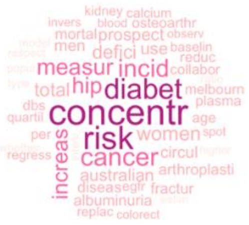	concentr*<br>risk<br>diabet*<br>cancer<br>incid*

	10.2337/dc10-2167. (60)

Note: The citation network provides an overview of the top 40 citation links within the topic. Each node is labelled with the surname of the first author of the citation, and the lines linking the nodes represents the citation links. The publication year is plotted on the left. The word cloud shows the frequency words within the topic and sub-topics, where words with a higher frequency have a larger size and opacity.

Supplementary Table S8. Topic 8: 14 publications, Vitamin D status and knee osteoarthritis

Citation Network	Word cloud

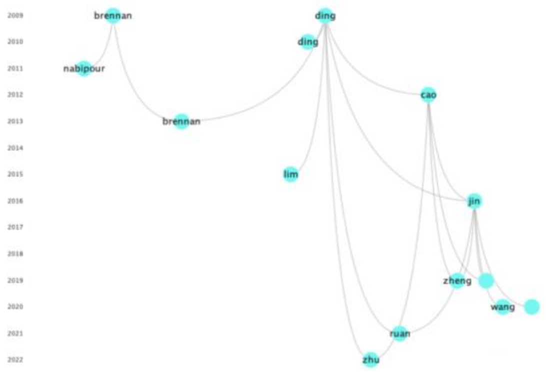

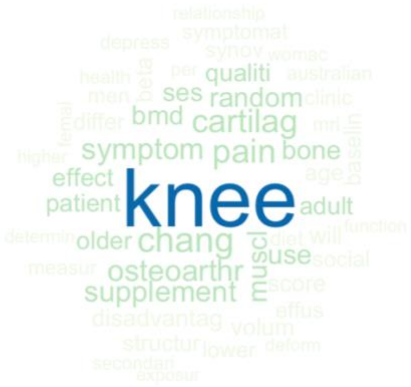

Indicative publication	Most unique topic terms
Ding C, Cicuttini F, Parameswaran V, Burgess J, Quinn S, Jones G. Serum levels of vitamin D, sunlight exposure, and knee cartilage loss in older adults: the Tasmanian older adult cohort study. Arthritis Rheum. 2009;60(5):1381-1389. doi: 10.1002/art.24486. (61)	knee<br>chang*<br>pain<br>cartilag*<br>osteoarthr*

Note: The citation network provides an overview of the citation links within the topic. Each node is labelled with the surname of the first author of the citation, and the lines linking the nodes represents the citation links. The publication year is plotted on the left. The word cloud shows the frequency words within the topic and sub-topics, where words with a higher frequency have a larger size and opacity.

Supplementary Table S9. Topic 9: 7 publications, Vitamin D status and exercise performance in athletes

Citation Network	Word cloud
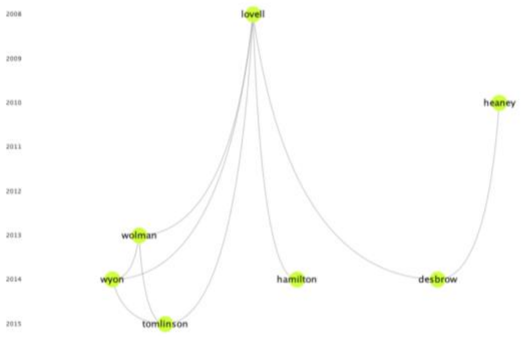	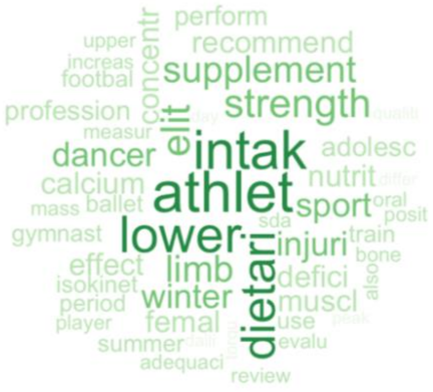

Indicative publication	Most unique topic terms
Lovell G. Vitamin D status of females in an elite gymnastics program. Clin J Sport Med. 2008;18(2):159-161. doi: 10.1097/JSM.0b013e3181650eee. (62)	athlet*<br>intak*<br>lower<br>dietari*<br>strength

Note: The citation network provides an overview of the citation links within the topic. Each node is labelled with the surname of the first author of the citation, and the lines linking the nodes represents the citation links. The publication year is plotted on the left. The word cloud shows the frequency words within the topic and sub-topics, where words with a higher frequency have a larger size and opacity.

References

1. Nowson CA, Margerison C. Vitamin D Intake and Vitamin D Status of Australians. *Med J Aust*. (2002) 177(3):149-52. doi: 10.5694/j.1326-5377.2002.tb04702.x.

2. Grover SR, Morley R. Vitamin D Deficiency in Veiled or Dark-Skinned Pregnant Women. *Med J Aust*. (2001) 175(5):251-2. doi: 10.5694/j.1326-5377.2001.tb143558.x.

3. Skull SA, Ngeow JY, Biggs BA, Street A, Ebeling PR. Vitamin D Deficiency Is Common and Unrecognized among Recently Arrived Adult Immigrants from the Horn of Africa. *Intern Med J*. (2003) 33(1-2):47-51. doi: 10.1046/j.1445-5994.2003.00344.x.

4. Nozza JM, Rodda CP. Vitamin D Deficiency in Mothers of Infants with Rickets. *Med J Aust*. (2001) 175(5):253-5. doi: 10.5694/j.1326-5377.2001.tb143559.x.

5. Joshi D, Center JR, Eisman JA. Vitamin D Deficiency in Adults. *Aust Prescr*. (2010) 33:103-6. doi: 10.18773/austprescr.2010.053.

6. Brock K, Wilkinson M, Cook R, Lee S, Bermingham M. Associations with Vitamin D Deficiency in "at Risk" Australians. *J Steroid Biochem Mol Biol*. (2004) 89-90(1-5):581-8. doi: 10.1016/j.jsbmb.2004.03.073.

7. Paxton GA, Teale GR, Nowson CA, Mason RS, McGrath JJ, Thompson MJ, et al. Vitamin D and Health in Pregnancy, Infants, Children and Adolescents in Australia and New Zealand: A Position Statement. *Med J Aust*. (2013) 198(3):142-3. doi: 10.5694/mja11.11592.

8. Bowyer L, Catling-Paull C, Diamond T, Homer C, Davis G, Craig ME. Vitamin D, Pth and Calcium Levels in Pregnant Women and Their Neonates. *Clin Endocrinol (Oxf)*. (2009) 70(3):372-7. doi: 10.1111/j.1365-2265.2008.03316.x.

9. Teale GR, Cunningham CE. Vitamin D Deficiency Is Common among Pregnant Women in Rural Victoria. *Aust N Z J Obstet Gynaecol*. (2010) 50(3):259-61. doi: 10.1111/j.1479-828X.2010.01147.x.

10. Thomas SD, Fudge AN, Whiting M, Coates PS. The Correlation between Third-Trimester Maternal and Newborn-Serum 25-Hydroxy-Vitamin D in a Selected South Australian Group of Newborn Samples. *BMJ Open*. (2011) 1(2):e000236. doi: 10.1136/bmjopen-2011-000236.

11. Munns CF, Simm PJ, Rodda CP, Garnett SP, Zacharin MR, Ward LM, et al. Incidence of Vitamin D Deficiency Rickets among Australian Children: An Australian Paediatric Surveillance Unit Study. *Med J Aust*. (2012) 196(7):466-8. doi: 10.5694/mja11.10662.

12. Matsuyama M, Harb T, David M, Davies PS, Hill RJ. Effect of Fortified Milk on Growth and Nutritional Status in Young Children: A Systematic Review and Meta-Analysis. *Public Health Nutr*. (2017) 20(7):1214-25. doi: 10.1017/s1368980016003189.

13. Rigterink T, Appleton L, Day AS. Vitamin D Therapy in Children with Inflammatory Bowel Disease: A Systematic Review. *World J Clin Pediatr*. (2019) 8(1):1-14. doi: 10.5409/wjcp.v8.i1.1.

14. Whitehouse AJO, Holt BJ, Serralha M, Holt PG, Kusel MMH, Hart PH. Maternal Serum Vitamin D Levels During Pregnancy and Offspring Neurocognitive Development. *Pediatrics*. (2012) 129(3):485-93. doi: 10.1542/peds.2011-2644.

15. Pasco JA, Henry MJ, Nicholson GC, Sanders KM, Kotowicz MA. Vitamin D Status of Women in the Geelong Osteoporosis Study: Association with Diet and Casual Exposure to Sunlight. *Med J Aust*. (2001) 175(8):401-5. doi: 10.5694/j.1326-5377.2001.tb143643.x.

16. Jones G, Dwyer T, Hynes KL, Parameswaran V, Greenaway TM. Vitamin D Insufficiency in Adolescent Males in Southern Tasmania: Prevalence, Determinants, and Relationship to Bone Turnover Markers. *Osteoporos Int*. (2005) 16(6):636-41. doi: 10.1007/s00198-004-1733-z.

17. Pasco JA, Wark JD, Carlin JB, Ponsonby AL, Vuillermin PJ, Morley R. Maternal Vitamin D in Pregnancy May Influence Not Only Offspring Bone Mass but Other Aspects of Musculoskeletal Health and Adiposity. *Med Hypotheses*. (2008) 71(2):266-9. doi: 10.1016/j.mehy.2008.01.033.

18. Tiong AC, Patel MS, Gardiner J, Ryan R, Linton KS, Walker KA, et al. Health Issues in Newly Arrived African Refugees Attending General Practice Clinics in Melbourne. *Med J Aust*. (2006) 185(11-12):602-6. doi: 10.5694/j.1326-5377.2006.tb00724.x.

19. van der Mei IA, Ponsonby AL, Dwyer T, Blizzard L, Taylor BV, Kilpatrick T, et al. Vitamin D Levels in People with Multiple Sclerosis and Community Controls in Tasmania, Australia. *J Neurol*. (2007) 254(5):581-90. doi: 10.1007/s00415-006-0315-8.

20. Lucas RM, Ponsonby AL, Dear K, Valery PC, Pender MP, Taylor BV, et al. Sun Exposure and Vitamin D Are Independent Risk Factors for Cns Demyelination. *Neurology*. (2011) 76(6):540-8. doi: 10.1212/WNL.0b013e31820af93d.

21. Lucas RM, Ponsonby AL, Dear K, Valery PC, Taylor B, van der Mei I, et al. Vitamin D Status: Multifactorial Contribution of Environment, Genes and Other Factors in Healthy Australian Adults across a Latitude Gradient. *J Steroid Biochem Mol Biol*. (2013) 136:300-8. doi: 10.1016/j.jsbmb.2013.01.011.

22. Lucas R, Ponsonby AL, McMichael A, van der Mei I, Chapman C, Coulthard A, et al. Observational Analytic Studies in Multiple Sclerosis: Controlling Bias through Study Design and Conduct. The Australian Multicentre Study of Environment and Immune Function. *Mult Scler*. (2007) 13(7):827-39. doi: 10.1177/1352458507077174.

23. Staples JA, Ponsonby A-L, Lim LL-Y, McMichael AJ. Ecologic Analysis of Some Immune-Related Disorders, Including Type 1 Diabetes, in Australia: Latitude, Regional Ultraviolet Radiation, and Disease Prevalence. *Environ Health Perspect*. (2003) 111(4):518-23. doi: 10.1289/ehp.5941.

24. Jablonski NG, Chaplin G. The Evolution of Human Skin Coloration. *J Hum Evol*. (2000) 39(1):57-106. doi: 10.1006/jhev.2000.0403.

25. van der Mei IA, Blizzard L, Ponsonby AL, Dwyer T. Validity and Reliability of Adult Recall of Past Sun Exposure in a Case-Control Study of Multiple Sclerosis. *Cancer Epidemiol Biomarkers Prev*. (2006) 15(8):1538-44. doi: 10.1158/1055-9965.Epi-05-0969.

26. Elliott JC, Lucas RM, Clements MS, Bambrick HJ. Population Density Determines the Direction of the Association between Ambient Ultraviolet Radiation and Type 1 Diabetes Incidence. *Pediatr Diabetes*. (2010) 11(6):394-402. doi: 10.1111/j.1399-5448.2009.00620.x.

27. Flicker L, Mead K, MacInnis RJ, Nowson C, Scherer S, Stein MS, et al. Serum Vitamin D and Falls in Older Women in Residential Care in Australia. *J Am Geriatr Soc*. (2003) 51(11):1533-8. doi: 10.1046/j.1532-5415.2003.51510.x.

28. Stein MS, Scherer SC, Walton SL, Gilbert RE, Ebeling PR, Flicker L, et al. Risk Factors for Secondary Hyperparathyroidism in a Nursing Home Population. *Clin Endocrinol (Oxf)*. (1996) 44(4):375-83. doi: 10.1046/j.1365-2265.1996.701521.x.

29. Sambrook PN, Cameron ID, Chen JS, Cumming RG, Durvasula S, Herrmann M, et al. Does Increased Sunlight Exposure Work as a Strategy to Improve Vitamin D Status in the Elderly: A Cluster Randomised Controlled Trial. *Osteoporos Int*. (2012) 23(2):615-24. doi: 10.1007/s00198-011-1590-5.

30. Nowson CA, Sherwin AJ, McPhee JG, Wark JD, Flicker L. Energy, Protein, Calcium, Vitamin D and Fibre Intakes from Meals in Residential Care Establishments in Australia. *Asia Pac J Clin Nutr*. (2003) 12(2):172-7.

31. Quaggiotto P, Tran H, Bhanugopan M. Vitamin D Deficiency Remains Prevalent Despite Increased Laboratory Testing in New South Wales, Australia. *Singapore Med J*. (2014) 55(5):271-80. doi: 10.11622/smedj.2014071.

32. Flicker L, MacInnis RJ, Stein MS, Scherer SC, Mead KE, Nowson CA, et al. Should Older People in Residential Care Receive Vitamin D to Prevent Falls? Results of a Randomized Trial. *J Am Geriatr Soc*. (2005) 53(11):1881-8. doi: 10.1111/j.1532-5415.2005.00468.x.

33. Sambrook PN, Chen JS, March LM, Cameron ID, Cumming RG, Lord SR, et al. Serum Parathyroid Hormone Predicts Time to Fall Independent of Vitamin D Status in a Frail Elderly Population. *J Clin Endocrinol Metab*. (2004) 89(4):1572-6. doi: 10.1210/jc.2003-031782.

34. Hirani V, Naganathan V, Cumming RG, Blyth F, Le Couteur DG, Handelsman DJ, et al. Associations between Frailty and Serum 25-Hydroxyvitamin D and 1,25-Dihydroxyvitamin D Concentrations in Older Australian Men: The Concord Health and Ageing in Men Project. *J Gerontol A Biol Sci Med Sci*. (2013) 68(9):1112-21. doi: 10.1093/gerona/glt059.

35. Latham NK, Anderson CS, Lee A, Bennett DA, Moseley A, Cameron ID. A Randomized, Controlled Trial of Quadriceps Resistance Exercise and Vitamin D in Frail Older People: The Frailty Interventions Trial in Elderly Subjects (Fitness). *J Am Geriatr Soc*. (2003) 51(3):291-9. doi: 10.1046/j.1532-5415.2003.51101.x.

36. Morris HA, Morrison GW, Burr M, Thomas DW, Nordin BE. Vitamin D and Femoral Neck Fractures in Elderly South Australian Women. *Med J Aust*. (1984) 140(9):519-21. doi: 10.5694/j.1326-5377.1984.tb108222.x.

37. Daly RM, Gagnon C, Lu ZX, Magliano DJ, Dunstan DW, Sikaris KA, et al. Prevalence of Vitamin D Deficiency and Its Determinants in Australian Adults Aged 25 Years and Older: A National, Population-Based Study. *Clin Endocrinol (Oxf)*. (2012) 77(1):26-35. doi: 10.1111/j.1365-2265.2011.04320.x.

38. Gagnon C, Lu ZX, Magliano DJ, Dunstan DW, Shaw JE, Zimmet PZ, et al. Low Serum 25-Hydroxyvitamin D Is Associated with Increased Risk of the Development of the Metabolic Syndrome at Five Years: Results from a National, Population-Based Prospective Study (the Australian Diabetes, Obesity and Lifestyle Study: Ausdiab). *J Clin Endocrinol Metab*. (2012) 97(6):1953-61. doi: 10.1210/jc.2011-3187.

39. Black LJ, Burrows SA, Jacoby P, Oddy WH, Beilin LJ, Chan She Ping-Delfos W, et al. Vitamin D Status and Predictors of Serum 25-Hydroxyvitamin D Concentrations in Western Australian Adolescents. *Br J Nutr*. (2014) 112(7):1154-62. doi: 10.1017/s000711451400186x.

40. Malacova E, Cheang PR, Dunlop E, Sherriff JL, Lucas RM, Daly RM, et al. Prevalence and Predictors of Vitamin D Deficiency in a Nationally Representative Sample of Adults Participating in the 2011-2013 Australian Health Survey. *Br J Nutr*. (2019) 121(8):894-904. doi: 10.1017/s0007114519000151.

41. Vanlint SJ, Morris HA, Newbury JW, Crockett AJ. Vitamin D Insufficiency in Aboriginal Australians. *Med J Aust*. (2011) 194(3):131-4. doi: 10.5694/j.1326-5377.2011.tb04195.x.

42. Dunlop E, Cunningham J, Sherriff JL, Lucas RM, Greenfield H, Arcot J, et al. Vitamin D_3 and 25-Hydroxyvitamin D_3 Content of Retail White Fish and Eggs in Australia. *Nutrients*. (2017) 9(7):647. doi: 10.3390/nu9070647.

43. Black LJ, Jacoby P, Nowson CA, Daly RM, Lucas RM. Predictors of Vitamin D-Containing Supplement Use in the Australian Population and Associations between Dose and Serum 25-Hydroxyvitamin D Concentrations. *Nutrients*. (2016) 8(6):356. doi: 10.3390/nu8060356.

44. Hughes AM, Armstrong BK, Vajdic CM, Turner J, Grulich AE, Fritschi L, et al. Sun Exposure May Protect against Non-Hodgkin Lymphoma: A Case-Control Study. *Int J Cancer*. (2004) 112(5):865-71. doi: 10.1002/ijc.20470.

45. van der Mei IA, Ponsonby AL, Engelsen O, Pasco JA, McGrath JJ, Eyles DW, et al. The High Prevalence of Vitamin D Insufficiency across Australian Populations Is Only Partly Explained by Season and Latitude. *Environ Health Perspect*. (2007) 115(8):1132-9. doi: 10.1289/ehp.9937.

46. Bogh MKB, Schmedes AV, Philipsen PA, Thieden E, Wulf HC. Vitamin D Production after Uvb Exposure Depends on Baseline Vitamin D and Total Cholesterol but Not on Skin Pigmentation. *J Invest Dermatol*. (2010) 130(2):546-53. doi: 10.1038/jid.2009.323.

47. Youl PH, Janda M, Kimlin M. Vitamin D and Sun Protection: The Impact of Mixed Public Health Messages in Australia. *Int J Cancer*. (2009) 124(8):1963-70. doi: 10.1002/ijc.24154.

48. Marks R, Foley PA, Jolley D, Knight KR, Harrison J, Thompson SC. The Effect of Regular Sunscreen Use on Vitamin D Levels in an Australian Population. Results of a Randomized Controlled Trial. *Arch Dermatol*. (1995) 131(4):415-21. doi: 10.1001/archderm.1995.01690160043006.

49. Kimlin M, Harrison S, Nowak M, Moore M, Brodie A, Lang C. Does a High Uv Environment Ensure Adequate Vitamin D Status? *J Photochem Photobiol B*. (2007) 89(2-3):139-47. doi: 10.1016/j.jphotobiol.2007.09.008.

50. Jayaratne N, Russell A, van der Pols JC. Sun Protection and Vitamin D Status in an Australian Subtropical Community. *Prev Med*. (2012) 55(2):146-50. doi: 10.1016/j.ypmed.2012.05.011.

51. Godar DE. Uv Doses Worldwide. *Photochem Photobiol*. (2005) 81(4):736-49. doi: 10.1562/2004-09-07-ir-308r.1.

52. Pasco JA, Henry MJ, Kotowicz MA, Sanders KM, Seeman E, Pasco JR, et al. Seasonal Periodicity of Serum Vitamin D and Parathyroid Hormone, Bone Resorption, and Fractures: The Geelong Osteoporosis Study. *J Bone Miner Res*. (2004) 19(5):752-8. doi: 10.1359/jbmr.040125.

53. Pasco JA, Henry MJ, Nicholson GC, Brennan SL, Kotowicz MA. Behavioural and Physical Characteristics Associated with Vitamin D Status in Women. *Bone*. (2009) 44(6):1085-91. doi: 10.1016/j.bone.2009.02.020.

54. Callegari ET, Reavley N, Garland SM, Gorelik A, Wark JD. Vitamin D Status, Bone Mineral Density and Mental Health in Young Australian Women: The Safe-D Study. *J Public Health Res*. (2015) 4(3):594. doi: 10.4081/jphr.2015.594.

55. Sambrook PN, Seeman E, Phillips SR, Ebeling PR. Preventing Osteoporosis: Outcomes of the Australian Fracture Prevention Summit. *Med J Aust*. (2002) 176(S8):S1-16. doi: 10.5694/j.1326-5377.2002.tb04475.x.

56. Ebeling PR, Daly RM, Kerr DA, Kimlin MG. Building Healthy Bones Throughout Life: An Evidence-Informed Strategy to Prevent Osteoporosis in Australia. *Med J Aust*. (2013) 199(S7):S1-S46. doi: 10.5694/j.1326-5377.2013.tb04225.x.

57. Nguyen TV, Center JR, Eisman JA. Osteoporosis: Underrated, Underdiagnosed and Undertreated. *Med J Aust*. (2004) 180(S5):S18-22. doi: 10.5694/j.1326-5377.2004.tb05908.x.

58. Bleicher K, Naganathan V, Cumming RG, Seibel MJ, Sambrook PN, Blyth FM, et al. Prevalence and Treatment of Osteoporosis in Older Australian Men: Findings from the Champ Study. *Med J Aust*. (2010) 193(7):387-91. doi: 10.5694/j.1326-5377.2010.tb03964.x.

59. Bilinski K, Boyages S. Evidence of Overtesting for Vitamin D in Australia: An Analysis of 4.5 Years of Medicare Benefits Schedule (Mbs) Data. *BMJ Open*. (2013) 3(6):e002955. doi: 10.1136/bmjopen-2013-002955.

60. Gagnon C, Lu ZX, Magliano DJ, Dunstan DW, Shaw JE, Zimmet PZ, et al. Serum 25-Hydroxyvitamin D, Calcium Intake, and Risk of Type 2 Diabetes after 5 Years: Results from a National, Population-Based Prospective Study (the Australian Diabetes, Obesity and Lifestyle Study). *Diabetes Care*. (2011) 34(5):1133-8. doi: 10.2337/dc10-2167.

61. Ding C, Cicuttini F, Parameswaran V, Burgess J, Quinn S, Jones G. Serum Levels of Vitamin D, Sunlight Exposure, and Knee Cartilage Loss in Older Adults: The Tasmanian Older Adult Cohort Study. *Arthritis Rheum*. (2009) 60(5):1381-9. doi: 10.1002/art.24486.

62. Lovell G. Vitamin D Status of Females in an Elite Gymnastics Program. *Clin J Sport Med*. (2008) 18(2):159-61. doi: 10.1097/JSM.0b013e3181650eee.
